# Correction: Study protocol for a randomised trial for atosiban versus placebo in threatened preterm birth: the APOSTEL 8 study

**DOI:** 10.1136/bmjopen-2019-029101corr1

**Published:** 2022-10-21

Klumper J, Breebaart W, Roos C, *et al*. Study protocol for a randomised trial for atosiban versus placebo in threatened preterm birth: the APOSTEL 8 study. *BMJ Open* 2019;9:e029101. doi: 10.1136/bmjopen-2019-029101

The article has been corrected since it was published online. The authors would like to notify on the following changes done in the paper.

Under the heading **Abstract**.

**Old text:** A sample size of 1514 participants (757 per group) will detect a reduction in adverse neonatal outcome from 10% to 6% (alpha 0.05, beta 0.2).

**New text:** A sample size of 760 participants (380 per group) will detect a reduction in adverse neonatal outcome from 11.95% to 6% (alpha error 0.05, beta error 0.2).

Under the heading **Monitoring and safety**.

**Old text:** A formal interim analysis is planned after data collection of 500 and of 1000 women.

**New text:** A formal interim analysis is planned after data collection of 500 women.

Under the heading **Sample size**.

**Old text:** Based on the APOSTEL three data, the proportion of adverse perinatal outcome in women randomised between 30 and 34 weeks’ gestation and treated with atosiban was 6%.^10^ To show a 40% reduction (10% in the placebo group to 6% in the atosiban group), we need to randomise 1438 women (beta error 0.2; alpha error 0.05). Assuming a 5% drop-out or loss-to follow-up rate, we will randomise 1514 women (757 in each arm).

**New text:** Based on the APOSTEL three data, the proportion of adverse perinatal outcome in women randomised between 30 and 34 weeks gestation and treated with atosiban was 6%.^10^ Based on two recent studies,^27,28^ we expect a 49.8% reduction of 11.95% adverse perinatal outcome in the placebo group to 6% in the atosiban group. Therefore, we need to randomise 722 women (beta-error 0.2; alpha error 0.05). Assuming a 5% drop-out rate, we need to randomise 760 women (380 in each arm).

**New references**

27. Lorthe E, Goffinet F, Marret S, *et al*. Tocolysis after preterm premature rupture of membranes and neonatal outcome: a propensity-score analysis. *Am J Obstet Gynecol*. 2017;217(2):212.e1–212.e12.

28. van Winden TMS, Roos C, Nijman T a. J, *et al*. Tocolysis compared with no tocolysis in women with threatened preterm birth and ruptured membranes: A propensity score analysis. *Eur J Obstet Gynecol Reprod Biol*. 2020;255:67–73

