# Supplementary material for: Study protocol for a randomised trial for atosiban versus placebo in threatened preterm birth: the APOSTEL 8 study
Source: BMJ Open. 2019 Nov 26;9(11):e029101. doi: 10.1136/bmjopen-2019-029101 (PMC6887072; doi:10.1136/bmjopen-2019-029101)
Supplement: Author's manuscript [file bmjopen-2019-029101.draft_revisions.pdf]

BMJ Open is committed to open peer review. As part of this commitment we make the peer review history of every article we publish publicly available.

When an article is published we post the peer reviewers' comments and the authors' responses online. We also post the versions of the paper that were used during peer review. These are the versions that the peer review comments apply to.

The versions of the paper that follow are the versions that were submitted during the peer review process. They are not the versions of record or the final published versions. They should not be cited or distributed as the published version of this manuscript.

BMJ Open is an open access journal and the full, final, typeset and author-corrected version of record of the manuscript is available on our site with no access controls, subscription charges or pay-per-view fees (http://bmjopen.bmj.com).

If you have any questions on BMJ Open's open peer review process please email info.bmjopen@bmj.com

BMJ Open

Study protocol for a randomised trial for atosiban versus placebo in threatened preterm birth: the APOSTEL 8 study

Journal:	*BMJ Open*
Manuscript ID	bmjopen-2019-029101
Article Type:	Protocol
Date Submitted by the Author:	14-Jan-2019
Complete List of Authors:	Klumper, Job; Amsterdam UMC, location AMC, Department of obstetrics & gynaecology<br>Breebaart, Wouter; Amsterdam UMC, location AMC, Department of obstetrics & gynaecology<br>Roos, Carolien; Amsterdam UMC, location AMC, Department of obstetrics & gynaecology<br>Naaktgeboren, CA ; Julius Centrum voor Gezondheidswetenschappen en Eerstelijns Geneeskunde, University Medical Centre Utrecht<br>van der Post, Joris; Amsterdam UMC, location AMC, Department of obstetrics & gynaecology<br>Bosmans, J; Vrije Universiteit Amsterdam, Department of Health Sciences, Amsterdam Public Health research institute<br>van Kaam, Anton; Amsterdam UMC, location AMC and VUmc, Department of neonatology<br>Schuit, Ewoud; Julius Center for Health Sciences and Primary Care, University Medical Centre Utrecht; Stanford University, Standford Prevention Research Center<br>Mol, Ben; School of Medicine, Monash University, Melbourne, Australia, Department of obstetrics & gynaecology<br>Baalman, Jelle; National Maternity Hospital, Department of obstetrics & gynaecology<br>McAuliffe, Fionnuala; University College Dublin, UCD Perinatal Research Centre; National Maternity Hospital, Department of obstetrics & gynaecology<br>Thornton, Jim; University of Nottingham, Department of obstetrics & gynaecology<br>Kok, Marjolein; Amsterdam UMC, location AMC, Department of obstetrics & gynaecology<br>Oudijk, Martijn; Amsterdam UMC, location AMC, Department of obstetrics & gynaecology
Keywords:	preterm birth, preterm labour, tocolysis, atosiban, perinatal outcome

SCHOLARONE™
Manuscripts

Study protocol for a randomised trial for atosiban versus placebo in threatened preterm birth: the APOSTEL 8 study

J. Klumper,[1] W. Breebaart,[1] C. Roos,[1] C.A. Naaktgeboren,[2] J.A.M van der Post,[1] J.E. Bosmans,[3] A.H. van Kaam,[4] E. Schuit,[2] B.W. Mol,[5] J.H. Baalman,[6] F.M. McAuliffe,[6] J.G. Thornton,[8] M. Kok[1] and M.A. Oudijk.[1]

Affiliations

[1]Department of Obstetrics and Gynaecology, Amsterdam UMC, University of Amsterdam, The Netherlands

[2]Julius Centre for Health Sciences and Primary Care, University Medical Centre Utrecht, Utrecht University, the Netherlands

[3]Department of Health Sciences, Faculty of Science, Vrije Universiteit Amsterdam, Amsterdam Public Health research institute, The Netherlands

[4]Department of Neonatology, Amsterdam UMC, Vrije Universiteit van Amsterdam, University of Amsterdam, the Netherlands.

[5]The Robinson Institute, School of Paediatrics and Reproductive Health, University of Adelaide, Australia.

[6]UCD Perinatal Research Centre, School of Medicine, University College Dublin, National Maternity Hospital, Dublin, Ireland

[7]Department of Child Health Obstetrics and Gynaecology, Nottingham University Hospitals NHS Trust, University of Nottingham, United Kingdom

Corresponding author

M.A. Oudijk, MD, PhD

Email: m.a.oudijk@amc.nl

Amsterdam University Medical Centres, location AMC

PO box 22660 | 1100 DD Amsterdam

Telephone: +31 20 5667204

W. Breebaart: breebaart@gmail.com

C. Roos: c.dijkstra@amc.uva.nl

C.A. Naaktgeboren: c.naaktgeboren@umcutrecht.nl

J.A.M van der Post: j.a.vanderpost@amc.uva.nl

J.E. Bosmans: j.e.bosmans@vu.nl

A.H. van Kaam: a.h.vankaam@amc.uva.nl

E. Schuit: e.schuit@umcutrecht.nl

B.W. Mol: ben.mol@monash.edu

J.H. Baalman: jelle.baalman@gmail.com

F.M. McAuliffe: fionnuala.mcauliffe@ucd.ie

J.G. Thornton: jim.thornton@nottingham.ac.uk

M.A. Oudijk: m.a.oudijk@amc.uva.nl

M. Kok: m.kok@amc.uva.nl

For peer review only

Abstract

Introduction: Preterm birth complicates more than 15 million pregnancies annually worldwide. In many countries, women who present with signs of preterm labour are treated with tocolytics for 48 hours. Although this delays birth, it has never been shown to improve neonatal outcome. In 2015 the WHO stated that the use of tocolytics should be reconsidered and that large placebo controlled studies to evaluate the effectiveness of tocolytics are urgently needed.

Methods and analysis: An international multicentre, randomised, double blinded, placebo-controlled clinical trial.

Participants: Women with threatened preterm birth (gestational age 30 – 34 weeks) defined as uterine contractions with

1) a cervical length of ≤ 15 mm or

2) a cervical length of 15-30 mm and a positive fibronectin test or

3) in centres where cervical length measurement is not part of the local protocol: a positive fibronectin test or insulin-like growth factor binding protein-1 (Actim-Partus test) or

4) ruptured membranes.

Intervention: Atosiban infusion for 48 hours

Control: placebo infusion for 48 hours

Primary outcome: A composite of perinatal mortality and severe neonatal morbidity.

Analysis: Analysis will be by intention to treat. A sample size of 1514 participants (757 per group) will detect a reduction in adverse neonatal outcome from 10% to 6% (alpha 0.05, beta 0.2). A cost-effectiveness analysis will be performed from a societal perspective.

Ethics and dissemination: The Ethics Committee of the Amsterdam University Medical Centres, location AMC, has approved this study. The results will be presented at conferences and published in a peer-reviewed journal. Participants will be informed about the results.

Discussion: This trial will show whether tocolysis with atosiban reduces adverse neonatal outcome in women with threatened preterm birth at 30-34 weeks gestation.

Trial registration number: NTR6646 (date registration 24-aug-2017)

Keywords: preterm birth, preterm labour, tocolysis, atosiban, perinatal outcome

Article summary

Strengths and limitations of this study:

For peer review only

- The primary outcome is perinatal mortality and neonatal morbidity, not prolongation of pregnancy.
- This is the largest randomised trial comparing atosiban to placebo for women with threatened preterm birth.
- Over 40 hospitals in Europe will participate.
- Tocolysis is incorporated in daily routine as it has been the recommendation in many guidelines. This will prove to be a challenge in counselling patients to participate in a placebo controlled trial, especially in an acute setting.

Introduction

Preterm birth, defined as birth before 37 weeks gestation, is a major contributor to perinatal mortality and morbidity, complicating over 15 million pregnancies worldwide.[1,2] Of all infant deaths before the age of 5 years, more than one third can be attributed to preterm birth.[3] In addition, spontaneous preterm birth is the leading cause of neonatal morbidity, mostly due to respiratory immaturity, intracranial haemorrhage and infections.[4,5] These conditions can have long-term neurodevelopmental sequelae such as cognitive impairment, cerebral palsy and visual and hearing deficiencies. Preterm birth is one of the largest single contributors to the Global Burden of Disease because of the high mortality early in life and the morbidity of lifelong impairment.[6]

Maternal administration of corticosteroids to accelerate fetal lung maturation is an effective treatment for women with threatened preterm birth.[7] Since steroids have their maximum effect if birth is delayed by 48 hours, many obstetricians administer a tocolytic drug alongside the steroids to allow maximal steroid effect and facilitate transport of the mother to a centre with neonatal intensive care unit facilities if needed. Several tocolytics are used, including β adrenoceptor agonists, cyclooxygenase inhibitors (COX), magnesium sulphate, calcium-channel blockers and oxytocin receptor antagonists. Though more or less effective in delaying delivery, no tocolytics used in obstetrical practice are proven effective in reducing neonatal morbidity and mortality.[8,9] The two most commonly used tocolytic drugs, atosiban and nifedipine, showed comparable perinatal outcome in the APOSTEL III study.[10] However, neonatal mortality was higher in the nifedipine group, although not significant (5.4% vs. 2.4% RR 2.20; 95% CI 0.91-5.33).

The oxytocin receptor antagonist atosiban has fewer maternal side effects in head to head comparison with alternative drugs.[11] In placebo controlled trials, atosiban has not shown a reduction in perinatal mortality (RR 2.25, 95% CI 0.79 to 6.38; two studies with 729 infants) or major neonatal morbidity.[12]

For peer review only

One explanation might be that since spontaneous preterm birth is associated in 40-70% of cases with chorioamnionitis,[13,14] tocolysis may prolong fetal exposure to an infectious environment, which may worsen neonatal outcome.

Perinatal outcome has also markedly improved over the last few decades, in part due to postnatal interventions such as exogenous surfactant treatment which reduces mortality and respiratory morbidity in preterm infants.[15] This might also limit the potential benefit of tocolytics.

Worldwide, practice varies widely. Several large institutions in countries like Canada, Scotland and Ireland, rarely use tocolytics, while in the USA, cyclooxygenase inhibitors (indomethacin) and calcium channel blockers (nifedipine) are popular. In Europe, nifedipine and the oxytocin antagonist, atosiban, are both widely used.

In conclusion, current widespread use of tocolytic drugs for this indication is not supported by the available evidence. The primary goal of tocolysis should not be prolongation of pregnancy, but improvement of neonatal outcome. This view is supported by the WHO, as they state in their 2015 guidelines on preterm birth that the effectiveness of tocolytics is not proven, and that placebo controlled studies are urgently needed.[16] Based on the results of the APOSTEL III study,[10] the associated editorial,[17] and its safety profile we chose to evaluate atosiban in the APOSTEL 8 study.

Objective

To test the hypothesis that tocolysis with atosiban in late preterm birth (30 to 34 weeks) reduces neonatal mortality and morbidity and is cost-effective compared with placebo.

Methods and analysis

Design and setting

We will conduct an international, multicentre, double-blind, randomised, placebo-controlled clinical trial, performed in The Netherlands, Belgium, United Kingdom and Ireland.

Participants/eligibility criteria

Women, aged ≥18 years, with threatened preterm birth and a gestational age between 30 $^{+0}$ and 33 $^{+6}$ weeks are eligible. Threatened preterm birth is defined as uterine contractions with 1) a cervical length of ≤ 15 mm or

For peer review only

2) a cervical length of 15-30 mm and a positive fibronectin test or
3) Or in centres where cervical length measurement is not part of the local protocol: a positive fibronectin test or insulin-like growth factor binding protein-1 (Actim-Partus test) or
4) ruptured membranes.

Both women with singleton and multiple pregnancies are eligible.

Women with a contra-indication for tocolysis, signs of fetal distress, clinical signs of intra-uterine infection, previous treatment for threatened preterm birth with corticosteroids in the current pregnancy and known fetal chromosomal or severe structural abnormalities are not eligible.

Procedures, recruitment, randomization and collection of data

Potential participants will be identified by the local research co-ordinators and/or the staff of participating hospitals. Women eligible for the trial will be counselled by doctors, midwifes or research nurses trained in 'good clinical practice', and will be given a patient information form to read. Those who wish to participate, will be asked to give for written informed consent, and are registered within the central trial database. Randomisation will be performed by using sequentially numbered medication packs available in each centre. Only the independent data manager has access to the computer-generated randomization list in which the medication numbers are linked with atosiban or placebo. Treatment allocation is blinded to investigators, participants, clinicians and research coordinators. Randomisation will be balanced with varying block sizes of 2 and 4, and stratified by centre.

At study entry, baseline demographic, past obstetric and medical history will be recorded into the web-based Case Report Form (CRF) accessible through a secure central website (Castor Electronic Data Capture, Ciwit B.V.)[18] Details of delivery, maternal and neonatal assessments during pregnancy and post-partum period will be recorded on the same system. All data will be coded, processed and stored with adequate precautions to ensure patient confidentially.

Interventions

Participants are allocated to atosiban or matching placebo (0.9% saline) for 48 hours. The medication will be administered by a bolus injection of 6.75 mg/0.9 ml in one minute followed by a continuous infusion of 18 mg/hour for 3 hours followed by a continuous infusion of 6 mg/hour for the remaining 45 hours. Participating women will otherwise be treated according to local protocol based on national guidelines, including corticosteroids and antibiotics if needed.

For peer review only

Outcome measures

Outcome parameters are in line with the core outcome set for studies on prevention of preterm birth defined by members of GONet and the Core Outcomes in Women's health (CROWN) initiative (www.crown-initiative.org).[19]

The primary outcome measure is a composite of adverse perinatal outcome composed of perinatal in-hospital mortality and six severe perinatal morbidities: bronchopulmonary (BPD) dysplasia at 36 weeks postmenstrual age, periventricular leucomalacia (PVL) > grade 1, intraventricular haemorrhage > grade 2, necrotizing enterocolitis (NEC) ≥ stage 2, retinopathy of prematurity > grade 2 or needing laser therapy, and culture proven sepsis. The diagnosis of BPD will be made according to the international consensus guideline as described by Jobe and Bancalari.[20] PVL > grade 1 and intraventricular haemorrhage > grade 2 will be diagnosed by repeated cranial ultrasound according to the guidelines on neuro imaging described by de Vries et al.[21] and Ment et al.[22] NEC ≥ stage 2 will be diagnosed according to Bell.[23] Culture proven sepsis is diagnosed on the combination of clinical signs and positive blood cultures. The components of the composite adverse perinatal outcome will also be assessed separately.

Secondary infant outcomes will be birth within 48 hours, time to birth, gestational age at birth, birth weight, number of days on invasive mechanical ventilation, length of NICU stay, convulsions, asphyxia, meningitis, pneumothorax until hospital discharge.

Maternal outcomes will be mortality, infection of inflammation, prelabor rupture of membranes and harm to mother from interventions (side effects). Side effects are defined as admission to intensive care, anaphylactic shock, dyspnoea, hypotension (leading to CTG abnormalities), liver test abnormalities (elevated ASAT or ALAT), general side effects (nausea, vomiting, headache), post-partum haemorrhage defined as > 500 ml blood loss and maternal mortality.

We will ask informed consent to approach the parents for long-term follow-up of the children. We intend, subject to funding, to use standardized questionnaires at 2 years and 5 years of age.

Maternal quality of life will be assessed at randomisation and at three months baby corrected age using the EuroQol (EQ-5D-5L) questionnaire. This consists of five dimensions (mobility, self-care, usual activities, pain/discomfort, anxiety/depression) that are rated using five levels (no problems, slight problems, moderate problems, severe problems, extreme problems).

For peer review only

Societal costs will be assessed using adapted versions of the iMTA Medical and Productivity Cost Questionnaires at 40 weeks postmenstrual age and 3 months corrected baby age. Cost data include costs of the intervention, other health care utilization, patient and family costs and costs of productivity losses.

Withdrawal of subjects

Participants can cease study treatment at any time for any reason if they wish to do so. Unless they refuse to allow further data collection, such participants will continue to be followed-up and will be analysed in the group to which they were originally allocated. Participants who decline follow-up will have no further trial data collected. Any results collected up to the point at which they decline follow up will be analysed. Study medication will be discontinued in patients with signs of intra-uterine infections or signs of fetal distress (abnormal CTG, meconium stained amniotic fluid). Data of such participants will continue to be analysed. Further management will be left to the expertise of the responsible clinician. The responsible clinician can contact a perinatologist from the project group in case of suspected side effects or other medical problems. If necessary, treatment will be discontinued.

Monitoring and Safety

An independent data safety monitoring board (DSMB) will focus on both effectiveness and safety. Serious Adverse Events (SAEs) will be collected from the first study-related procedure until 3 months after delivery. All SAEs will be reported to the DSMB within 7 days. Whenever there is proof of effectiveness (at interim analysis) or safety issues (increased (serious) adverse events in one of the two treatment arms) the DSMB will advise whether the trial should be stopped or continued. The data safety monitoring board will be blinded when first analysing the data, but unblinded before reaching a decision.
The advice(s) of the DSMB will only be sent to the sponsor of the study. Should the sponsor decide not to fully implement the advice of the DSMB, the sponsor will send the advice to the reviewing Medical Ethics Committee, including a note to substantiate why (part of) the advice of the DSMB will not be followed.

A formal interim analysis is planned after data collection of 500 and of 1000 women. At these interim analyses, the Haybittle-Peto alpha spending function will be used, which means that an effect at interim with a p-value <0.001 is considered statistically significant.

Sample size

For peer review only

Based on the APOSTEL III data, the proportion of adverse perinatal outcome in women randomized between 30 and 34 weeks gestation and treated with atosiban was 6%.[10] To show a 40% reduction (10% in the placebo group to 6% in the atosiban group) we need to randomise 1438 women (beta-error 0.2; alpha error 0.05). Assuming a 5% drop-out or loss-to follow up rate, we will randomize 1514 women (757 in each arm).

Statistical analysis

Data analysis

Data analysis will be performed according to the intention-to-treat principle. In the baseline table, categorical variables will be expressed as a number with the percentage of the total allocation arm. Continuous variables will be presented as mean with standard deviation, as geometric mean with 95% confidence interval (CI) or as median with interquartile range, whichever appropriate.

The main outcome 'adverse neonatal outcome' will be assessed on the infant level, using a log-binomial generalized estimating equations model (GEEs), resulting in a relative risk (RR) with accompanying 95% confidence interval (CI). To account for stratified randomization by centre, we will also take centre into the model if the model converges.[24] We will account for interdependence between outcomes in multiple pregnancies by considering the mother as a cluster variable.[25]

The other secondary outcome measures on the child level will be analyzed similarly to the primary outcome measure. Outcomes on the maternal level will be assessed by using a binomial regression model with log-link function. When a statistically significant difference in primary outcome is found between both groups, we will calculate the number needed to treat (NNT). Time to delivery will be evaluated by Kaplan-Meier estimates and Cox proportional hazard analysis, taking into account the different durations of gestation at study entry, and will be tested with the log rank test. A p-value of less than 0.05 will be considered to indicate statistical significance.

Subgroup analyses

The following subgroup analyses are planned:

1) singleton versus multiple pregnancy,

2) cervical length < 15 mm, versus cervical length 15 - 30 mm and a positive fibronectin test (or no cervical length measurement and a positive Fibronectin test or Partus test),

3) ruptured or unruptured membranes at entry

For peer review only

5) previous preterm birth.

Sensitivity analysis

A sensitivity analysis will be performed excluding multiple pregnancies and pregnancies complicated by preterm premature rupture of membranes.

To assess whether a subgroup effect is present we will add an interaction term between the subgrouping variables and the treatment allocation to the regression model. When an interaction term is statistically significant ($p<0.05$), we will estimate the treatment effect within strata of the subgrouping variable.

Details of the statistical analysis will be describes in separate statistical analysis plan that will be completed before data lock.

Cost-effectiveness analysis

The cost-effectiveness analysis will be done according to the intention-to treat principle. Missing cost and effect data will be imputed using multiple imputation according to the MICE algorithm developed by van Buuren.[26] Rubin's rules will be used to pool the results from the different multiply imputed datasets. Bivariate regression analyses will be used to estimate cost and effect differences between atosiban and placebo while adjusting for confounders if necessary. Incremental cost-effectiveness ratios (ICERs) will be calculated by dividing the difference in the mean total costs between the treatment groups by the difference in mean effect between the treatment groups. Bias-corrected and accelerated bootstrapping with 5000 replications will be used to estimate statistical uncertainty. Uncertainty surrounding ICERs will be graphically presented on cost-effectiveness planes. Cost-effectiveness acceptability curves will be estimated showing the probability that atosiban is cost-effective in comparison with placebo for a range of different ceiling ratios thereby showing decision uncertainty. Sensitivity analyses will be performed to assess the robustness of the results using different assumptions regarding costs and effects.

For peer review only

Patient and Public Involvement

The preterm birth research line of the Dutch Consortium is in close collaboration with two Dutch patient associations, the VOC (Vereniging van Ouders van Couveusekinderen, freely translated to society of parents of children admitted to NICU) and the NVOM (Nederlandse Vereniging van Ouders van Meerlingen, freely translated to Dutch society of parents of multiples). They are involved in the design of new studies, updated on progress of running trials, and informed of study results. Project members are invited speakers at yearly conferences of these societies to present on the progress of our preterm birth research line. At these conferences, surveys are being performed on patient preferences on study ideas.

Tocolysis was deemed an important research issue. Both associations have written support letters to the funding agency ZonMw (The Netherlands organization for health research and development) for the APOSTEL 8 study.

A project panel of parents that experienced a spontaneous preterm birth consisting of 6 couples was involved in the design of our study. A survey was performed during the design of the study amongst members of the closed Facebook group of the VOC, to address questions on whether they would be interested in participation in the APOSTEL 8 study.

The Dutch consortium has a website where it publishes all results of completed studies, and publishes the protocols of currently recruiting studies.

Presentations will be held at yearly conferences at patient organizations and updates on research are being published in the journal of the VOC.

For peer review only

Ethics and dissemination

The Medical Ethics Committee at the Amsterdam University Medical Centres, location AMC, approved this study. Additional regional approval was obtained for the remaining participating hospitals in The Netherlands. For Ireland and United Kingdom, both national and local authorities approved this trial according to national regulations.

This trial is registered with the Nederlands Trial Register, NTR6646.

A manuscript with the results of the primary study will be published in a peer-reviewed journal. A separate manuscript will be written on the cost effectiveness analysis.

The results of this clinical trial will be presented at conferences and disseminated through publication in a peer-reviewed journal.

Acknowledgements

We would like to thank the collaborators of the study group; the gynecologists of the participating centres for their help as local investigators for the APOSTEL 8 study.

We also thank the patient associations VOC and NVOW for their input in this study.

Author contributions

CAN, JAMP, JEB, AHLCvK, BWM, JHB, FMcA, JGT, MAO, MK were involved in conception and design of the study.

JK and WB drafted the manuscript.

CR, CAN, JAMP, JEB, AHLCvK, BWM, JHB, FMcA, JGT, MAO, MK reviewed and edited the manuscript. All authors mentioned in the manuscript are member of the APOSTEL study

For peer review only - http://bmjopen.bmj.com/site/about/guidelines.xhtml

group or collaborators. They participated in the design of the study during several meetings and are local investigators in the participating centres. All authors edited the manuscript and read and approved the final manuscript.

Funding

This study is funded by ZonMw (The Netherlands organization for health research and development), grant number 848041004 and the United Kingdom National Institute for Health Research, Clinical Research Network.

Competing interests statement

JGT received a number of lectures and medical advisory fees from Ferring pharmaceuticals between 2000 and 2016.

References

1. Howson CP, Kinney MV, Lawn JE (editors). Born Too Soon: The Global Action Report on Preterm Birth. Geneva: World Health Organization, 2012
2. Liu L, Johnson HL, Cousens S, Perin J, Scott S, Lawn JE, et al. Global, regional, and national causes of child mortality: an updated systematic analysis for 2010 with time trends since 2000. Lancet 2012;379(9832):2151–61
3. Matthews TJ, Macdorman MF, Thoma ME. Infant Mortality Statistics From the 2013 Period Linked Birth/Infant Death Data Set. Natl Vital Stat Rep. 2015;64(9):1-30
4. Hack M, Fanaroff AA. Outcomes of children of extremely low birthweight and gestational age in the 1990's. Early Hum Dev. 1999;53(3):193-218.
5. Moore T, Hennessy EM, Myles J, et al. Neurological and developmental outcome in extremely preterm children born in England in 1995 and 2006: the EPICure studies. BMJ. 2012;345:e7961.
6. Lozano R, Naghavi M, Foreman K, et al. Global and regional mortality from 235 causes of death for 20 age groups in 1990 and 2010: a systematic analysis for the Global Burden of Disease Study 2010. Lancet. 2012;380(9859):2095-128.
7. Roberts D, Brown J, Medley N, Dalziel SR. Antenatal corticosteroids for accelerating fetal lung maturation for women at risk of preterm birth. Cochrane Database Syst Rev. 2017;3:CD004454.
8. Haas DM, Imperiale TF, Kirkpatrick PR, Klein RW, Zollinger TW, Golichowski AM. Tocolytic therapy: a meta-analysis and decision analysis. Obstet Gynecol 2009; 113: 585–94.

For peer review only

9. Haas DM, Caldwell DM, Kirkpatrick P, McIntosh JJ, Welton NJ. Tocolytic therapy for preterm delivery: systematic review and network meta-analysis. BMJ 2012; 345: e6226.
10. Van Vliet EO, Nijman TA, Schuit E, et al. Nifedipine versus atosiban for threatened preterm birth (APOSTEL III): a multicentre, randomised controlled trial. Lancet. 2016;387(10033):2117-24.
11. De Heus R, Mol BW, Erwich JJ, et al. Adverse drug reactions to tocolytic treatment for preterm labour: prospective cohort study. BMJ. 2009;338:b744.
12. Flenady V, Reinebrant HE, Liley HG, Tambimuttu EG, Papatsonis DNM. Oxytocin receptor antagonists for inhibiting pretermlabour. Cochrane Database of Systematic Reviews 2014, Issue 6. Art. No.: CD004452.
13. Tita AT, Andrews WW. Diagnosis and management of clinical chorioamnionitis. Clin Perinatol. 2010;37(2):339-54.
14. Romero R, Dey SK, Fisher SJ. Preterm labor: one syndrome, many causes. Science. 2014;345(6198):760-5.
15. Soll R, Ozek E. Prophylactic protein free synthetic surfactant for preventing morbidity and mortality in preterm infants. Cochrane Database Syst Rev. 2010;(1):CD001079.
16. WHO (2015, November 17). WHO recommendation on the use of tocolytic treatment for inhibiting preterm labour. Retrieved from: *https://extranet.who.int/rhl/topics/preconception-pregnancy-childbirth-and-postpartum-care/pregnancy-complications/preterm-birth/who-recommendation-use-tocolytic-treatment-inhibiting-preterm-labour*
17. Walker KF, Thornton JG. Tocolysis and preterm labour. Lancet. 2016;387(10033):2068-2070.
18. Castor: Amsterdam, Ciwit BV the N. Castor electronic data capture. 2017.
19. van 't Hooft J, Duffy JM, Daly M, Williamson P, Meher S, Thom E, et al. A core outcome Set for evaluation of interventions to prevent preterm birth. Obstet Gynecol. 2016;127(1):49–58.
20. Jobe AH, Bancalari E: Bronchopulmonary dysplasia. Am J Respir Crit Care. Med 2001, 163:1723–1729.
21. de Vries LS, Eken P, Dubowitz LM: The spectrum of leukomalacia using cranial ultrasound. Behav Brain Res 1992, 49:1–6.
22. Ment LR, Bada HS, Barnes P, Grant PE, Hirtz D, Papile LA, Pinto-Martin J, Rivkin M, Slovis TL: Practice parameter: neuroimaging of the neonate: report of the Quality Standards Subcommittee of the American Academy of Neurology and the Practice Committee of the Child Neurology Society. Neurology 2002, 58(12):1726–1738.
23. Bell MJ: Neonatal necrotizing enterocolitis. Ann Surg 1978, 187:1–7.

For peer review only

398 24. Kahan BC. Accounting for centre-effects in multicentre trials with a binary outcome - when, why, and how?. BMC Med Res Methodol. 2014;14:20.

400 25. Gates S, Brocklehurst P. How should randomised trials including multiple pregnancies be analysed?. BJOG. 2004;111(3):213-9.

402 26. Van Buuren, S., and Groothuis-Oudshoorn, K. (2011). MICE: Multivariate Imputation by Chained Equations in R. *Journal of Statistical Software* 45(3), 1-68

404 ## Word count

405 2611 words

For peer review only

SPIRIT

STANDARD PROTOCOL ITEMS: RECOMMENDATIONS FOR INTERVENTIONAL TRIALS

SPIRIT 2013 Checklist: Recommended items to address in a clinical trial protocol and related documents*

Section/item	Item No	Description
**Administrative information**		
Title	1	Descriptive title identifying the study design, population, interventions, and, if applicable, trial acronym
Trial registration	2a	Trial identifier and registry name. If not yet registered, name of intended registry
	2b	All items from the World Health Organization Trial Registration Data Set
Protocol version	3	Date and version identifier
Funding	4	Sources and types of financial, material, and other support
Roles and responsibilities	5a	Names, affiliations, and roles of protocol contributors
	5b	Name and contact information for the trial sponsor
	5c	Role of study sponsor and funders, if any, in study design; collection, management, analysis, and interpretation of data; writing of the report; and the decision to submit the report for publication, including whether they will have ultimate authority over any of these activities
	5d	Composition, roles, and responsibilities of the coordinating centre, steering committee, endpoint adjudication committee, data management team, and other individuals or groups overseeing the trial, if applicable (see Item 21a for data monitoring committee)
**Introduction**		
Background and rationale	6a	Description of research question and justification for undertaking the trial, including summary of relevant studies (published and unpublished) examining benefits and harms for each intervention
	6b	Explanation for choice of comparators
Objectives	7	Specific objectives or hypotheses
Trial design	8	Description of trial design including type of trial (eg, parallel group, crossover, factorial, single group), allocation ratio, and framework (eg, superiority, equivalence, noninferiority, exploratory)

For peer review only

Methods: Participants, interventions, and outcomes

Study setting	9	Description of study settings (eg, community clinic, academic hospital) and list of countries where data will be collected. Reference to where list of study sites can be obtained
Eligibility criteria	10	Inclusion and exclusion criteria for participants. If applicable, eligibility criteria for study centres and individuals who will perform the interventions (eg, surgeons, psychotherapists)
Interventions	11a	Interventions for each group with sufficient detail to allow replication, including how and when they will be administered
	11b	Criteria for discontinuing or modifying allocated interventions for a given trial participant (eg, drug dose change in response to harms, participant request, or improving/worsening disease)
	11c	Strategies to improve adherence to intervention protocols, and any procedures for monitoring adherence (eg, drug tablet return, laboratory tests)
	11d	Relevant concomitant care and interventions that are permitted or prohibited during the trial
Outcomes	12	Primary, secondary, and other outcomes, including the specific measurement variable (eg, systolic blood pressure), analysis metric (eg, change from baseline, final value, time to event), method of aggregation (eg, median, proportion), and time point for each outcome. Explanation of the clinical relevance of chosen efficacy and harm outcomes is strongly recommended
Participant timeline	13	Time schedule of enrolment, interventions (including any run-ins and washouts), assessments, and visits for participants. A schematic diagram is highly recommended (see Figure)
Sample size	14	Estimated number of participants needed to achieve study objectives and how it was determined, including clinical and statistical assumptions supporting any sample size calculations
Recruitment	15	Strategies for achieving adequate participant enrolment to reach target sample size

Methods: Assignment of interventions (for controlled trials)

Allocation:

Sequence generation	16a	Method of generating the allocation sequence (eg, computer-generated random numbers), and list of any factors for stratification. To reduce predictability of a random sequence, details of any planned restriction (eg, blocking) should be provided in a separate document that is unavailable to those who enrol participants or assign interventions

For peer review only - http://bmjopen.bmj.com/site/about/guidelines.xhtml

Allocation concealment mechanism	16b	Mechanism of implementing the allocation sequence (eg, central telephone; sequentially numbered, opaque, sealed envelopes), describing any steps to conceal the sequence until interventions are assigned
Implementation	16c	Who will generate the allocation sequence, who will enrol participants, and who will assign participants to interventions
Blinding (masking)	17a	Who will be blinded after assignment to interventions (eg, trial participants, care providers, outcome assessors, data analysts), and how
	17b	If blinded, circumstances under which unblinding is permissible, and procedure for revealing a participant's allocated intervention during the trial
**Methods: Data collection, management, and analysis**		
Data collection methods	18a	Plans for assessment and collection of outcome, baseline, and other trial data, including any related processes to promote data quality (eg, duplicate measurements, training of assessors) and a description of study instruments (eg, questionnaires, laboratory tests) along with their reliability and validity, if known. Reference to where data collection forms can be found, if not in the protocol
	18b	Plans to promote participant retention and complete follow-up, including list of any outcome data to be collected for participants who discontinue or deviate from intervention protocols
Data management	19	Plans for data entry, coding, security, and storage, including any related processes to promote data quality (eg, double data entry; range checks for data values). Reference to where details of data management procedures can be found, if not in the protocol
Statistical methods	20a	Statistical methods for analysing primary and secondary outcomes. Reference to where other details of the statistical analysis plan can be found, if not in the protocol
	20b	Methods for any additional analyses (eg, subgroup and adjusted analyses)
	20c	Definition of analysis population relating to protocol non-adherence (eg, as randomised analysis), and any statistical methods to handle missing data (eg, multiple imputation)
**Methods: Monitoring**		
Data monitoring	21a	Composition of data monitoring committee (DMC); summary of its role and reporting structure; statement of whether it is independent from the sponsor and competing interests; and reference to where further details about its charter can be found, if not in the protocol. Alternatively, an explanation of why a DMC is not needed

For peer review only - http://bmjopen.bmj.com/site/about/guidelines.xhtml

	21b	Description of any interim analyses and stopping guidelines, including who will have access to these interim results and make the final decision to terminate the trial
Harms	22	Plans for collecting, assessing, reporting, and managing solicited and spontaneously reported adverse events and other unintended effects of trial interventions or trial conduct
Auditing	23	Frequency and procedures for auditing trial conduct, if any, and whether the process will be independent from investigators and the sponsor
**Ethics and dissemination**		
Research ethics approval	24	Plans for seeking research ethics committee/institutional review board (REC/IRB) approval
Protocol amendments	25	Plans for communicating important protocol modifications (eg, changes to eligibility criteria, outcomes, analyses) to relevant parties (eg, investigators, REC/IRBs, trial participants, trial registries, journals, regulators)
Consent or assent	26a	Who will obtain informed consent or assent from potential trial participants or authorised surrogates, and how (see Item 32)
	26b	Additional consent provisions for collection and use of participant data and biological specimens in ancillary studies, if applicable
Confidentiality	27	How personal information about potential and enrolled participants will be collected, shared, and maintained in order to protect confidentiality before, during, and after the trial
Declaration of interests	28	Financial and other competing interests for principal investigators for the overall trial and each study site
Access to data	29	Statement of who will have access to the final trial dataset, and disclosure of contractual agreements that limit such access for investigators
Ancillary and post-trial care	30	Provisions, if any, for ancillary and post-trial care, and for compensation to those who suffer harm from trial participation
Dissemination policy	31a	Plans for investigators and sponsor to communicate trial results to participants, healthcare professionals, the public, and other relevant groups (eg, via publication, reporting in results databases, or other data sharing arrangements), including any publication restrictions
	31b	Authorship eligibility guidelines and any intended use of professional writers
	31c	Plans, if any, for granting public access to the full protocol, participant-level dataset, and statistical code

For peer review only

Appendices

Informed consent materials	32	Model consent form and other related documentation given to participants and authorised surrogates
Biological specimens	33	Plans for collection, laboratory evaluation, and storage of biological specimens for genetic or molecular analysis in the current trial and for future use in ancillary studies, if applicable

*It is strongly recommended that this checklist be read in conjunction with the SPIRIT 2013 Explanation & Elaboration for important clarification on the items. Amendments to the protocol should be tracked and dated. The SPIRIT checklist is copyrighted by the SPIRIT Group under the Creative Commons "Attribution-NonCommercial-NoDerivs 3.0 Unported" license.

For peer review only

BMJ Open

Study protocol for a randomised trial for atosiban versus placebo in threatened preterm birth: the APOSTEL 8 study

Journal:	*BMJ Open*
Manuscript ID	bmjopen-2019-029101.R1
Article Type:	Protocol
Date Submitted by the Author:	11-Jun-2019
Complete List of Authors:	Klumper, Job; Amsterdam UMC, location AMC, Department of obstetrics & gynaecology<br>Breebaart, Wouter; Amsterdam UMC, location AMC, Department of obstetrics & gynaecology<br>Roos, Carolien; Amsterdam UMC, location AMC, Department of obstetrics & gynaecology<br>Naaktgeboren, CA ; Julius Centrum voor Gezondheidswetenschappen en Eerstelijns Geneeskunde, University Medical Centre Utrecht<br>van der Post, Joris; Amsterdam UMC, location AMC, Department of obstetrics & gynaecology<br>Bosmans, J; Vrije Universiteit Amsterdam, Department of Health Sciences, Amsterdam Public Health research institute<br>van Kaam, Anton; Amsterdam UMC, location AMC and VUmc, Department of neonatology<br>Schuit, Ewoud; Julius Center for Health Sciences and Primary Care, University Medical Centre Utrecht; Stanford University, Standford Prevention Research Center<br>Mol, Ben; School of Medicine, Monash University, Melbourne, Australia, Department of obstetrics & gynaecology<br>Baalman, Jelle; National Maternity Hospital, Department of obstetrics & gynaecology<br>McAuliffe, Fionnuala; University College Dublin, UCD Perinatal Research Centre; National Maternity Hospital, Department of obstetrics & gynaecology<br>Thornton, Jim; University of Nottingham, Department of obstetrics & gynaecology<br>Kok, Marjolein; Amsterdam UMC, location AMC, Department of obstetrics & gynaecology<br>Oudijk, Martijn; Amsterdam UMC, location AMC, Department of obstetrics & gynaecology
**Primary Subject Heading**:	Obstetrics and gynaecology
Secondary Subject Heading:	Obstetrics and gynaecology
Keywords:	preterm birth, preterm labour, tocolysis, atosiban, perinatal outcome

SCHOLARONE™
Manuscripts

Study protocol for a randomised trial for atosiban versus placebo in threatened preterm birth: the APOSTEL 8 study

J. Klumper,[1] W. Breebaart,[1] C. Roos,[1] C.A. Naaktgeboren,[2] J.A.M van der Post,[1] J.E. Bosmans,[3] A.H. van Kaam,[4] E. Schuit,[2] B.W. Mol,[5] J.H. Baalman,[6] F.M. McAuliffe,[6] J.G. Thornton,[8] M. Kok[1] and M.A. Oudijk.[1]

Affiliations

[1]Department of Obstetrics and Gynaecology, Amsterdam UMC, University of Amsterdam, The Netherlands

[2]Julius Centre for Health Sciences and Primary Care, University Medical Centre Utrecht, Utrecht University, the Netherlands

[3]Department of Health Sciences, Faculty of Science, Vrije Universiteit Amsterdam, Amsterdam Public Health research institute, The Netherlands

[4]Department of Neonatology, Amsterdam UMC, Vrije Universiteit van Amsterdam, University of Amsterdam, the Netherlands.

[5]The Robinson Institute, School of Paediatrics and Reproductive Health, University of Adelaide, Australia.

[6]UCD Perinatal Research Centre, School of Medicine, University College Dublin, National Maternity Hospital, Dublin, Ireland

[7]Department of Child Health Obstetrics and Gynaecology, Nottingham University Hospitals NHS Trust, University of Nottingham, United Kingdom

Corresponding author

M.A. Oudijk, MD, PhD

Email: m.a.oudijk@amsterdamumc.nl

Amsterdam University Medical Centres, location AMC

PO box 22660 | 1100 DD Amsterdam

Telephone: +31 20 5667204

J. Klumper: j.klumper@amc.uva.nl

W. Breebaart: breebaart@gmail.com

C. Roos: c.dijkstra@amc.nl

C.A. Naaktgeboren: c.naaktgeboren@umcutrecht.nl

J.A.M van der Post: j.a.vanderpost@amc.uva.nl

J.E. Bosmans: j.e.bosmans@vu.nl

A.H. van Kaam: a.h.vankaam@amc.uva.nl

E. Schuit: e.schuit@umcutrecht.nl

B.W. Mol: ben.mol@monash.edu

J.H. Baalman: jelle.baalman@gmail.com

F.M. McAuliffe: fionnuala.mcauliffe@ucd.ie

J.G. Thornton: jim.thornton@nottingham.ac.uk

M. Kok: m.kok@amc.uva.nl

For peer review only

Abstract

Introduction: Preterm birth complicates more than 15 million pregnancies annually worldwide. In many countries, women who present with signs of preterm labour are treated with tocolytics for 48 hours. Although this delays birth, it has never been shown to improve neonatal outcome. In 2015 the WHO stated that the use of tocolytics should be reconsidered and that large placebo controlled studies to evaluate the effectiveness of tocolytics are urgently needed.

Methods and analysis: An international multicentre, randomised, double blinded, placebo-controlled clinical trial.

Participants: Women with threatened preterm birth (gestational age 30 – 34 weeks) defined as uterine contractions with

1) a cervical length of ≤ 15 mm or
2) a cervical length of 15-30 mm and a positive fibronectin test or
3) in centres where cervical length measurement is not part of the local protocol: a positive fibronectin test or insulin-like growth factor binding protein-1 (Actim-Partus test) or
4) ruptured membranes.

Intervention: Atosiban infusion for 48 hours

Control: placebo infusion for 48 hours

Primary outcome: A composite of perinatal mortality and severe neonatal morbidity.

Analysis: Analysis will be by intention to treat. A sample size of 1514 participants (757 per group) will detect a reduction in adverse neonatal outcome from 10% to 6% (alpha 0.05, beta 0.2). A cost-effectiveness analysis will be performed from a societal perspective.

Ethics and dissemination: The Research Ethics Committee (REC)of the Amsterdam University Medical Centres, location AMC, has approved this study. The study is currently under review by the local REC in Dublin, and the REC in the United Kingdom. The results will be presented at conferences and published in a peer-reviewed journal. Participants will be informed about the results.

Discussion: This trial will show whether tocolysis with atosiban reduces adverse neonatal outcome in women with threatened preterm birth at 30-34 weeks gestation.

Trial registration number: NTR6646 (date registration 24-aug-2017)

Protocol version: 2.0, dated 27-02-2019

Keywords: preterm birth, preterm labour, tocolysis, atosiban, perinatal outcome

Article summary

For peer review only

Strengths and limitations of this study:

- The primary outcome is perinatal mortality and neonatal morbidity, not prolongation of pregnancy.
- This is the largest randomised trial comparing atosiban to placebo for women with threatened preterm birth.
- Over 40 hospitals in Europe will participate.
- Tocolysis is incorporated in daily routine as it has been the recommendation in many guidelines.
- It will prove to be a challenge in counselling patients to participate in a placebo controlled trial, especially in an acute setting.

Introduction

Preterm birth, defined as birth before 37 weeks gestation, is a major contributor to perinatal mortality and morbidity, complicating over 15 million pregnancies worldwide.[1,2] Of all infant deaths before the age of 5 years, more than one third can be attributed to preterm birth.[3] In addition, spontaneous preterm birth is the leading cause of neonatal morbidity, mostly due to respiratory immaturity, intracranial haemorrhage and infections.[4,5] These conditions can have long-term neurodevelopmental sequelae such as cognitive impairment, cerebral palsy and visual and hearing deficiencies. Preterm birth is one of the largest single contributors to the Global Burden of Disease because of the high mortality early in life and the morbidity of lifelong impairment.[6]

Maternal administration of corticosteroids to accelerate fetal lung maturation is an effective treatment for women with threatened preterm birth.[7] Since steroids have their maximum effect if birth is delayed by 48 hours, many obstetricians administer a tocolytic drug alongside the steroids to allow maximal steroid effect and facilitate transport of the mother to a centre with neonatal intensive care unit facilities if needed. Several tocolytics are used, including β adrenoceptor agonists, cyclooxygenase inhibitors (COX), magnesium sulphate, calcium-channel blockers and oxytocin receptor antagonists. Though more or less effective in delaying delivery, no tocolytics used in obstetrical practice are proven effective in reducing neonatal morbidity and mortality.[8,9] None of the studies so far have been powered to show such an effect.

The two most commonly used tocolytic drugs, atosiban and nifedipine, showed comparable perinatal outcome in the APOSTEL III study.[10] However, neonatal mortality was higher in the nifedipine group, although not significant (5.4% vs. 2.4% RR 2.20; 95% CI 0.91-5.33).

For peer review only

The oxytocin receptor antagonist atosiban has fewer maternal side effects in head to head comparison with alternative drugs,[11] and showed similar effectiveness in delaying birth compared to ritodrine.[12] In placebo controlled trials, a Cochrane review showed that atosiban did not reduce perinatal mortality (RR 2.25, 95% CI 0.79 to 6.38; two studies with 729 infants) or major neonatal morbidity[13], although the quality of this review has been questioned.[14]

One explanation might be that since spontaneous preterm birth is associated in 40-70% of cases with chorioamnionitis,[15,16] tocolysis may prolong fetal exposure to an infectious environment, which may worsen neonatal outcome.

Perinatal outcome has also markedly improved over the last few decades, in part due to postnatal interventions such as exogenous surfactant treatment which reduces mortality and respiratory morbidity in preterm infants.[17] This might also limit the potential benefit of tocolytics.

Worldwide, practice varies widely. Several large institutions in countries like Canada, Scotland and Ireland, rarely use tocolytics, while in the USA, cyclooxygenase inhibitors (indomethacin) and calcium channel blockers (nifedipine) are popular. In Europe, nifedipine and the oxytocin antagonist, atosiban, are both widely used.

In conclusion, current widespread use of tocolytic drugs for this indication is not supported by the available evidence. The primary goal of tocolysis should not be prolongation of pregnancy, but improvement of neonatal outcome. This view is supported by the WHO, as they state in their 2015 guidelines on preterm birth that the effectiveness of tocolytics is not proven, and that placebo controlled studies are urgently needed.[18] Based on the results of the APOSTEL III study,[10] the associated editorial,[19] and its safety profile we chose to evaluate atosiban in the APOSTEL 8 study.

Objective

To test the hypothesis that tocolysis with atosiban in late preterm birth (30 to 34 weeks) reduces neonatal mortality and morbidity and is cost-effective compared with placebo.

Methods and analysis

Design and setting

For peer review only

We will conduct an international, multicentre, double-blind, randomised, placebo-controlled clinical trial, performed in The Netherlands, United Kingdom and Ireland.

Participants/eligibility criteria

Women, aged ≥18 years, with threatened preterm birth and a gestational age between 30^{+0} and 33^{+6} weeks are eligible. Threatened preterm birth is defined as uterine contractions with
1) a cervical length of ≤ 15 mm or
2) a cervical length of 15-30 mm and a positive fibronectin test or
3) Or in centres where cervical length measurement is not part of the local protocol: a positive fibronectin test or insulin-like growth factor binding protein-1 (Actim-Partus test) or
4) ruptured membranes.

These inclusion criteria are based on the results of the APOSTEL I study.[20] Moreover, our previous APOSTEL III study showed that half of the women with these criteria deliver within seven days,[10] validating this definition of women at high risk for preterm birth.

Both women with singleton and multiple pregnancies are eligible.
Women with a contra-indication for tocolysis, signs of fetal distress, clinical signs of intra-uterine infection, previous treatment for threatened preterm birth with corticosteroids in the current pregnancy and known fetal chromosomal or severe structural abnormalities are not eligible.

Procedures, recruitment, randomization and collection of data

Potential participants will be identified by the local research co-ordinators and/or the staff of participating hospitals. Women eligible for the trial will be counselled by doctors, midwifes or research nurses trained in ‘good clinical practice’, and will be given a patient information form to read. Those who wish to participate, will be asked to give for written informed consent, and are registered within the central trial database. Randomisation will be performed by using sequentially numbered medication packs available in each centre. Only the independent data manager has access to the computer-generated randomization list in which the medication numbers are linked with atosiban or placebo. Treatment allocation is blinded to investigators, participants, clinicians and research coordinators. Randomisation will be balanced with varying block sizes of 2 and 4, and stratified by centre.

At study entry, baseline demographic, past obstetric and medical history will be recorded into the web-based Case Report Form (CRF) accessible through a secure central website (Castor Electronic Data Capture, Ciwit B.V.)[21] Details of delivery, maternal and neonatal assessments during pregnancy and post-partum period will be recorded on the same

For peer review only

system. All data will be coded, processed and stored with adequate precautions to ensure patient confidentially. This is described in a separate data management plan.

Interventions

Participants are allocated to atosiban or matching placebo (0.9% saline) for 48 hours. The medication will be administered by a bolus injection of 6.75 mg/0.9 ml in one minute followed by a continuous infusion of 18 mg/hour for 3 hours followed by a continuous infusion of 6 mg/hour for the remaining 45 hours. Participating women will otherwise be treated according to local protocol based on national guidelines, including corticosteroids and antibiotics if needed.

Outcome measures

Outcome parameters are in line with the core outcome set for studies on prevention of preterm birth defined by members of GONet and the Core Outcomes in Women's health (CROWN) initiative (www.crown-initiative.org).[22]

The primary outcome measure is a composite of adverse perinatal outcome composed of perinatal in-hospital mortality and six severe perinatal morbidities: bronchopulmonary (BPD), periventricular leucomalacia (PVL) > grade 1, intraventricular haemorrhage > grade 2, necrotizing enterocolitis (NEC) ≥ stage 2, retinopathy of prematurity > grade 2 or needing laser therapy, and culture proven sepsis.

The diagnosis of BPD will be made according to the international consensus guideline as described by Jobe and Bancalari.[23] PVL > grade 1 and intraventricular haemorrhage > grade 2 will be diagnosed by repeated cranial ultrasound according to the guidelines on neuro imaging described by de Vries et al.[24] and Ment et al.[25] NEC ≥ stage 2 will be diagnosed according to Bell.[26] Culture proven sepsis is diagnosed on the combination of clinical signs and positive blood cultures. The components of the composite adverse perinatal outcome will also be assessed separately.

Secondary infant outcomes will be birth within 48 hours, time to birth, gestational age at birth, birth weight, number of days on invasive mechanical ventilation, length of NICU stay, convulsions, asphyxia, meningitis, pneumothorax until hospital discharge.

Maternal outcomes will be mortality, infection of inflammation and harm to mother from interventions (side effects). Side effects are defined as admission to intensive care, anaphylactic shock, dyspnoea, hypotension (leading to CTG abnormalities), liver test abnormalities (elevated ASAT or ALAT), general side effects (nausea, vomiting, headache), post-partum haemorrhage defined as > 500 ml blood loss and maternal mortality.

For peer review only

We will ask informed consent to approach the parents for long-term follow-up of the children. We intend, subject to funding, to use standardized questionnaires at 2 years and 5 years of age.

Maternal quality of life will be assessed at randomisation and at three months baby corrected age using the EuroQol (EQ-5D-5L) questionnaire. This consists of five dimensions (mobility, self-care, usual activities, pain/discomfort, anxiety/depression) that are rated using five levels (no problems, slight problems, moderate problems, severe problems, extreme problems).

Societal costs will be assessed using adapted versions of the iMTA Medical and Productivity Cost Questionnaires at three months corrected baby age. Cost data include costs of the intervention, other health care utilization, patient and family costs and costs of productivity losses.

Withdrawal of subjects

Participants can cease study treatment at any time for any reason if they wish to do so. Unless they refuse to allow further data collection, such participants will continue to be followed-up and will be analysed in the group to which they were originally allocated. Participants who decline follow-up will have no further trial data collected. Any results collected up to the point at which they decline follow up will be analysed. Study medication will be discontinued in patients with signs of intra-uterine infections or signs of fetal distress (abnormal CTG, meconium stained amniotic fluid). Data of such participants will continue to be analysed. Further management will be left to the expertise of the responsible clinician. The responsible clinician can contact a perinatologist from the project group in case of suspected side effects or other medical problems. If necessary, treatment will be discontinued.

Monitoring and Safety

An independent data safety monitoring board (DSMB) will focus on both effectiveness and safety. Serious Adverse Events (SAEs) will be collected from the first study-related procedure until 3 months after delivery. All SAEs will be reported to the DSMB within 7 days. Whenever there is proof of effectiveness (at interim analysis) or safety issues (increased (serious) adverse events in one of the two treatment arms) the DSMB will advise whether the trial should be stopped or continued. The data safety monitoring board will be blinded when first analysing the data, but unblinded before reaching a decision.
The advice(s) of the DSMB will only be sent to the sponsor of the study. Should the sponsor decide not to fully implement the advice of the DSMB, the sponsor will send the advice to the

For peer review only

reviewing Medical Ethics Committee, including a note to substantiate why (part of) the advice of the DSMB will not be followed.

A formal interim analysis is planned after data collection of 500 and of 1000 women. At these interim analyses, the Haybittle-Peto alpha spending function will be used, which means that an effect at interim with a p-value <0.001 is considered statistically significant.

Sample size

Based on the APOSTEL III data, the proportion of adverse perinatal outcome in women randomized between 30 and 34 weeks gestation and treated with atosiban was 6%.[10] To show a 40% reduction (10% in the placebo group to 6% in the atosiban group) we need to randomise 1438 women (beta-error 0.2; alpha error 0.05). Assuming a 5% drop-out or loss-to follow up rate, we will randomize 1514 women (757 in each arm).

Statistical analysis

Data analysis

Data analysis will be performed according to the intention-to-treat principle. In the baseline table, categorical variables will be expressed as a number with the percentage of the total allocation arm. Continuous variables will be presented as mean with standard deviation, as geometric mean with 95% confidence interval (CI) or as median with interquartile range, whichever appropriate.

The main outcome ‘adverse neonatal outcome’ will be assessed on the infant level, using a log-binomial generalized estimating equations model (GEEs) to take into account the correlation of outcomes in multiples, resulting in a relative risk (RR) with accompanying 95% confidence interval (CI). To account for stratified randomization by centre, we will also take centre into the model if the model converges.[27] We will account for interdependence between outcomes in multiple pregnancies by considering the mother as a cluster variable.[28]

The other secondary outcome measures on the child level will be analyzed similarly to the primary outcome measure. Outcomes on the maternal level will be assessed by using a binomial regression model with log-link function. When a statistically significant difference in primary outcome is found between both groups, we will calculate the number needed to treat (NNT). Time to delivery will be evaluated by Kaplan-Meier estimates and Cox proportional hazard analysis, taking into account the different durations of gestation at study entry, and will be tested with the log rank test. A p-value of less than 0.05 will be considered to indicate statistical significance.

For peer review only

Subgroup analyses

The following subgroup analyses are planned:

1) singleton versus multiple pregnancy,

2) cervical length < 15 mm, versus cervical length 15 - 30 mm and a positive fibronectin test (or no cervical length measurement and a positive Fibronectin test or Partus test),

3) ruptured or unruptured membranes at entry

5) previous preterm birth.

Sensitivity analysis

A sensitivity analysis will be performed excluding multiple pregnancies and pregnancies complicated by preterm premature rupture of membranes.

To assess whether a subgroup effect is present we will add an interaction term between the subgrouping variables and the treatment allocation to the regression model. When an interaction term is statistically significant ($p<0.05$), we will estimate the treatment effect within strata of the subgrouping variable.

Details of the statistical analysis will be describes in separate statistical analysis plan that will be completed before data lock.

Cost-effectiveness analysis

The cost-effectiveness analysis will be done according to the intention-to treat principle. Missing cost and effect data will be imputed using multiple imputation according to the MICE algorithm developed by van Buuren.[29] Rubin's rules will be used to pool the results from the different multiply imputed datasets. Bivariate regression analyses will be used to estimate cost and effect differences between atosiban and placebo while adjusting for confounders if necessary. Incremental cost-effectiveness ratios (ICERs) will be calculated by dividing the difference in the mean total costs between the treatment groups by the difference in mean effect between the treatment groups. Bias-corrected and accelerated bootstrapping with 5000 replications will be used to estimate statistical uncertainty. Uncertainty surrounding ICERs will be graphically presented on cost-effectiveness planes. Cost-effectiveness acceptability curves will be estimated showing the probability that atosiban is cost-effective in comparison with placebo for a range of different ceiling ratios thereby showing decision uncertainty. Sensitivity analyses will be performed to assess the robustness of the results using different assumptions regarding costs and effects.

For peer review only

Patient and Public Involvement

The preterm birth research line of the Dutch Consortium is in close collaboration with two Dutch patient associations, the VOC (Vereniging van Ouders van Couveusekinderen, freely translated to society of parents of children admitted to NICU) and the NVOM (Nederlandse Vereniging van Ouders van Meerlingen, freely translated to Dutch society of parents of multiples). They are involved in the design of new studies, updated on progress of running trials, and informed of study results. Project members are invited speakers at yearly conferences of these societies to present on the progress of our preterm birth research line. At these conferences, surveys are being performed on patient preferences on study ideas. Tocolysis was deemed an important research issue. Both associations have written support letters to the funding agency ZonMw (The Netherlands organization for health research and development) for the APOSTEL 8 study.

A project panel of parents that experienced a spontaneous preterm birth consisting of 6 couples was involved in the design of our study. A survey was performed during the design of the study amongst members of the closed Facebook group of the VOC, to address questions on whether they would be interested in participation in the APOSTEL 8 study.

The Dutch consortium has a website where it publishes all results of completed studies, and publishes the protocols of currently recruiting studies.

Presentations will be held at yearly conferences at patient organizations and updates on research are being published in the journal of the VOC.

Ethics and dissemination

The Research Ethics Committee (REC) at the Amsterdam University Medical Centres, location AMC, approved this study. Additional regional approval was obtained for the remaining participating hospitals in The Netherlands. The study is currently under review by the local REC in Dublin, and the REC in the United Kingdom.

Protocol amendments will be communicated to a relevant parties.

This trial is registered with the Nederlands Trial Register, NTR6646.

A manuscript with the results of the primary study will be published in a peer-reviewed journal. A separate manuscript will be written on the cost effectiveness analysis.

The results of this clinical trial will be presented at conferences and disseminated through publication in a peer-reviewed journal.

Acknowledgements

For peer review only

We would like to thank the collaborators of the study group; the gynecologists of the participating centres for their help as local investigators for the APOSTEL 8 study. We also thank the patient associations VOC and NVOW for their input in this study.

Author contributions

CAN, JAMP, JEB, AHvK, ES, BWM, JHB, FMcA, JGT, MAO, MK were involved in conception and design of the study.

JK and WB drafted the manuscript.

CR, CAN, JAMP, JEB, AHvK, ES, BWM, JHB, FMcA, JGT, MAO, MK reviewed and edited the manuscript. All authors mentioned in the manuscript are member of the APOSTEL study group or collaborators. They participated in the design of the study during several meetings and are local investigators in the participating centres. All authors edited the manuscript and read and approved the final manuscript.

Funding

This study is funded by ZonMw (The Netherlands organization for health research and development), grant number 848041004 and the United Kingdom National Institute for Health Research, Clinical Research Network.

Competing interests statement

JGT received a number of lectures and medical advisory fees from Ferring pharmaceuticals between 2000 and 2016.

References

1. Howson CP, Kinney MV, Lawn JE (editors). Born Too Soon: The Global Action Report on Preterm Birth. Geneva: World Health Organization, 2012
2. Liu L, Johnson HL, Cousens S, Perin J, Scott S, Lawn JE, et al. Global, regional, and national causes of child mortality: an updated systematic analysis for 2010 with time trends since 2000. Lancet 2012;379(9832):2151–61
3. Matthews TJ, Macdorman MF, Thoma ME. Infant Mortality Statistics From the 2013 Period Linked Birth/Infant Death Data Set. Natl Vital Stat Rep. 2015;64(9):1-30
4. Hack M, Fanaroff AA. Outcomes of children of extremely low birthweight and gestational age in the 1990's. Early Hum Dev. 1999;53(3):193-218.

For peer review only

5. Moore T, Hennessy EM, Myles J, et al. Neurological and developmental outcome in extremely preterm children born in England in 1995 and 2006: the EPICure studies. BMJ. 2012;345:e7961.

6. Lozano R, Naghavi M, Foreman K, et al. Global and regional mortality from 235 causes of death for 20 age groups in 1990 and 2010: a systematic analysis for the Global Burden of Disease Study 2010. Lancet. 2012;380(9859):2095-128.

7. Roberts D, Brown J, Medley N, Dalziel SR. Antenatal corticosteroids for accelerating fetal lung maturation for women at risk of preterm birth. Cochrane Database Syst Rev. 2017;3:CD004454.

8. Haas DM, Imperiale TF, Kirkpatrick PR, Klein RW, Zollinger TW, Golichowski AM. Tocolytic therapy: a meta-analysis and decision analysis. Obstet Gynecol 2009; 113: 585–94.

9. Haas DM, Caldwell DM, Kirkpatrick P, McIntosh JJ, Welton NJ. Tocolytic therapy for preterm delivery: systematic review and network meta-analysis. BMJ 2012; 345: e6226.

10. Van Vliet EO, Nijman TA, Schuit E, et al. Nifedipine versus atosiban for threatened preterm birth (APOSTEL III): a multicentre, randomised controlled trial. Lancet. 2016;387(10033):2117-24.

11. De Heus R, Mol BW, Erwich JJ, et al. Adverse drug reactions to tocolytic treatment for preterm labour: prospective cohort study. BMJ. 2009;338:b744.

12. Effectiveness and safety of the oxytocin antagonist atosiban versus beta-adrenergic agonists in the treatment of preterm labour. The Worldwide Atosiban versus Beta-agonists Study Group. BJOG. 2001;108(2):133-42.

13. Flenady V, Reinebrant HE, Liley HG, Tambimuttu EG, Papatsonis DNM. Oxytocin receptor antagonists for inhibiting preterm labour. Cochrane Database of Systematic Reviews 2014, Issue 6. Art. No.: CD004452.

14. Lyndrup J, and Lamont RF The choice of a tocolytic for the treatment of preterm labor: a critical evaluation of nifedipine versus atosiban. Expert Opin. Investig. Drugs (2007) 16(6):843-853.

15. Tita AT, Andrews WW. Diagnosis and management of clinical chorioamnionitis. Clin Perinatol. 2010;37(2):339-54.

16. Romero R, Dey SK, Fisher SJ. Preterm labor: one syndrome, many causes. Science. 2014;345(6198):760-5.

17. Soll R, Ozek E. Prophylactic protein free synthetic surfactant for preventing morbidity and mortality in preterm infants. Cochrane Database Syst Rev. 2010;(1):CD001079.

18. WHO (2015, November 17). WHO recommendation on the use of tocolytic treatment for inhibiting preterm labour. Retrieved from:

For peer review only

https://extranet.who.int/rhl/topics/preconception-pregnancy-childbirth-and-postpartum-care/pregnancy-complications/preterm-birth/who-recommendation-use-tocolytic-treatment-inhibiting-preterm-labour

19. Walker KF, Thornton JG. Tocolysis and preterm labour. Lancet. 2016;387(10033):2068-2070.
20. Van Baaren GJ, Vis JY, Wilms FF, et al. Predictive value of cervical length measurement and fibronectin testing in threatened preterm labor. Obstet Gynecol. 2014;123(6):1185-92.
21. Castor: Amsterdam, Ciwit BV the N. Castor electronic data capture. 2017.
22. van 't Hooft J, Duffy JM, Daly M, Williamson P, Meher S, Thom E, et al. A core outcome Set for evaluation of interventions to prevent preterm birth. Obstet Gynecol. 2016;127(1):49–58.
23. Jobe AH, Bancalari E: Bronchopulmonary dysplasia. Am J Respir Crit Care. Med 2001, 163:1723–1729.
24. de Vries LS, Eken P, Dubowitz LM: The spectrum of leukomalacia using cranial ultrasound. Behav Brain Res 1992, 49:1–6.
25. Ment LR, Bada HS, Barnes P, Grant PE, Hirtz D, Papile LA, Pinto-Martin J, Rivkin M, Slovis TL: Practice parameter: neuroimaging of the neonate: report of the Quality Standards Subcommittee of the American Academy of Neurology and the Practice Committee of the Child Neurology Society. Neurology 2002, 58(12):1726–1738.
26. Bell MJ: Neonatal necrotizing enterocolitis. Ann Surg 1978, 187:1–7.
27. Kahan BC. Accounting for centre-effects in multicentre trials with a binary outcome - when, why, and how?. BMC Med Res Methodol. 2014;14:20.
28. Gates S, Brocklehurst P. How should randomised trials including multiple pregnancies be analysed?. BJOG. 2004;111(3):213-9.
29. Van Buuren, S., and Groothuis-Oudshoorn, K. (2011). MICE: Multivariate Imputation by Chained Equations in R. *Journal of Statistical Software* 45(3), 1-68

Word count

2984 words

For peer review only

STANDARD PROTOCOL ITEMS: RECOMMENDATIONS FOR INTERVENTIONAL TRIALS

SPIRIT 2013 Checklist: Recommended items to address in a clinical trial protocol and related documents*

Section/item	Item No	Description	Addressed on page number
**Administrative information**			
Title	1	Descriptive title identifying the study design, population, interventions, and, if applicable, trial acronym	1
Trial registration	2a	Trial identifier and registry name. If not yet registered, name of intended registry	2
	2b	All items from the World Health Organization Trial Registration Data Set	2
Protocol version	3	Date and version identifier	2
Funding	4	Sources and types of financial, material, and other support	11
Roles and responsibilities	5a	Names, affiliations, and roles of protocol contributors	11
	5b	Name and contact information for the trial sponsor	1
	5c	Role of study sponsor and funders, if any, in study design; collection, management, analysis, and interpretation of data; writing of the report; and the decision to submit the report for publication, including whether they will have ultimate authority over any of these activities	11
	5d	Composition, roles, and responsibilities of the coordinating centre, steering committee, endpoint adjudication committee, data management team, and other individuals or groups overseeing the trial, if applicable (see Item 21a for data monitoring committee)	11

For peer review only

Introduction

Background and rationale	6a	Description of research question and justification for undertaking the trial, including summary of relevant studies (published and unpublished) examining benefits and harms for each intervention	3
	6b	Explanation for choice of comparators	4
Objectives	7	Specific objectives or hypotheses	4
Trial design	8	Description of trial design including type of trial (eg, parallel group, crossover, factorial, single group), allocation ratio, and framework (eg, superiority, equivalence, noninferiority, exploratory)	5

Methods: Participants, interventions, and outcomes

Study setting	9	Description of study settings (eg, community clinic, academic hospital) and list of countries where data will be collected. Reference to where list of study sites can be obtained	5
Eligibility criteria	10	Inclusion and exclusion criteria for participants. If applicable, eligibility criteria for study centres and individuals who will perform the interventions (eg, surgeons, psychotherapists)	5
Interventions	11a	Interventions for each group with sufficient detail to allow replication, including how and when they will be administered	6
	11b	Criteria for discontinuing or modifying allocated interventions for a given trial participant (eg, drug dose change in response to harms, participant request, or improving/worsening disease)	7
	11c	Strategies to improve adherence to intervention protocols, and any procedures for monitoring adherence (eg, drug tablet return, laboratory tests)	NA
	11d	Relevant concomitant care and interventions that are permitted or prohibited during the trial	6
Outcomes	12	Primary, secondary, and other outcomes, including the specific measurement variable (eg, systolic blood pressure), analysis metric (eg, change from baseline, final value, time to event), method of aggregation (eg, median, proportion), and time point for each outcome. Explanation of the clinical relevance of chosen efficacy and harm outcomes is strongly recommended	6
Participant timeline	13	Time schedule of enrolment, interventions (including any run-ins and washouts), assessments, and visits for participants. A schematic diagram is highly recommended (see Figure)	6

Sample size	14	Estimated number of participants needed to achieve study objectives and how it was determined, including clinical and statistical assumptions supporting any sample size calculations	______8______
Recruitment	15	Strategies for achieving adequate participant enrolment to reach target sample size	______5______

Methods: Assignment of interventions (for controlled trials)

Allocation:

Sequence generation	16a	Method of generating the allocation sequence (eg, computer-generated random numbers), and list of any factors for stratification. To reduce predictability of a random sequence, details of any planned restriction (eg, blocking) should be provided in a separate document that is unavailable to those who enrol participants or assign interventions	______5______
Allocation concealment mechanism	16b	Mechanism of implementing the allocation sequence (eg, central telephone; sequentially numbered, opaque, sealed envelopes), describing any steps to conceal the sequence until interventions are assigned	______5______
Implementation	16c	Who will generate the allocation sequence, who will enrol participants, and who will assign participants to interventions	______5______
Blinding (masking)	17a	Who will be blinded after assignment to interventions (eg, trial participants, care providers, outcome assessors, data analysts), and how	______5______
	17b	If blinded, circumstances under which unblinding is permissible, and procedure for revealing a participant's allocated intervention during the trial	______5______

Methods: Data collection, management, and analysis

Data collection methods	18a	Plans for assessment and collection of outcome, baseline, and other trial data, including any related processes to promote data quality (eg, duplicate measurements, training of assessors) and a description of study instruments (eg, questionnaires, laboratory tests) along with their reliability and validity, if known. Reference to where data collection forms can be found, if not in the protocol	______5______
	18b	Plans to promote participant retention and complete follow-up, including list of any outcome data to be collected for participants who discontinue or deviate from intervention protocols	______7______

For peer review only - http://bmjopen.bmj.com/site/about/guidelines.xhtml

Data management	19	Plans for data entry, coding, security, and storage, including any related processes to promote data quality (eg, double data entry; range checks for data values). Reference to where details of data management procedures can be found, if not in the protocol	5, 6
Statistical methods	20a	Statistical methods for analysing primary and secondary outcomes. Reference to where other details of the statistical analysis plan can be found, if not in the protocol	8
	20b	Methods for any additional analyses (eg, subgroup and adjusted analyses)	9
	20c	Definition of analysis population relating to protocol non-adherence (eg, as randomised analysis), and any statistical methods to handle missing data (eg, multiple imputation)	8, 9
**Methods: Monitoring**			
Data monitoring	21a	Composition of data monitoring committee (DMC); summary of its role and reporting structure; statement of whether it is independent from the sponsor and competing interests; and reference to where further details about its charter can be found, if not in the protocol. Alternatively, an explanation of why a DMC is not needed	7, 8
	21b	Description of any interim analyses and stopping guidelines, including who will have access to these interim results and make the final decision to terminate the trial	7, 8
Harms	22	Plans for collecting, assessing, reporting, and managing solicited and spontaneously reported adverse events and other unintended effects of trial interventions or trial conduct	7
Auditing	23	Frequency and procedures for auditing trial conduct, if any, and whether the process will be independent from investigators and the sponsor	7
**Ethics and dissemination**			
Research ethics approval	24	Plans for seeking research ethics committee/institutional review board (REC/IRB) approval	10
Protocol amendments	25	Plans for communicating important protocol modifications (eg, changes to eligibility criteria, outcomes, analyses) to relevant parties (eg, investigators, REC/IRBs, trial participants, trial registries, journals, regulators)	10

Consent or assent	26a	Who will obtain informed consent or assent from potential trial participants or authorised surrogates, and how (see Item 32)	5
	26b	Additional consent provisions for collection and use of participant data and biological specimens in ancillary studies, if applicable	NA
Confidentiality	27	How personal information about potential and enrolled participants will be collected, shared, and maintained in order to protect confidentiality before, during, and after the trial	5, 6
Declaration of interests	28	Financial and other competing interests for principal investigators for the overall trial and each study site	11
Access to data	29	Statement of who will have access to the final trial dataset, and disclosure of contractual agreements that limit such access for investigators	6
Ancillary and post-trial care	30	Provisions, if any, for ancillary and post-trial care, and for compensation to those who suffer harm from trial participation	Not named
Dissemination policy	31a	Plans for investigators and sponsor to communicate trial results to participants, healthcare professionals, the public, and other relevant groups (eg, via publication, reporting in results databases, or other data sharing arrangements), including any publication restrictions	10
	31b	Authorship eligibility guidelines and any intended use of professional writers	NA
	31c	Plans, if any, for granting public access to the full protocol, participant-level dataset, and statistical code	NA
**Appendices**			
Informed consent materials	32	Model consent form and other related documentation given to participants and authorised surrogates	NA
Biological specimens	33	Plans for collection, laboratory evaluation, and storage of biological specimens for genetic or molecular analysis in the current trial and for future use in ancillary studies, if applicable	NA

*It is strongly recommended that this checklist be read in conjunction with the SPIRIT 2013 Explanation & Elaboration for important clarification on the items. Amendments to the protocol should be tracked and dated. The SPIRIT checklist is copyrighted by the SPIRIT Group under the Creative Commons "Attribution-NonCommercial-NoDerivs 3.0 Unported" license.

For peer review only - http://bmjopen.bmj.com/site/about/guidelines.xhtml

BMJ Open

Study protocol for a randomised trial for atosiban versus placebo in threatened preterm birth: the APOSTEL 8 study

Journal:	*BMJ Open*
Manuscript ID	bmjopen-2019-029101.R2
Article Type:	Protocol
Date Submitted by the Author:	07-Oct-2019
Complete List of Authors:	Klumper, Job; Amsterdam UMC, location AMC, Department of obstetrics & gynaecology<br>Breebaart, Wouter; Amsterdam UMC, location AMC, Department of obstetrics & gynaecology<br>Roos, Carolien; Amsterdam UMC, location AMC, Department of obstetrics & gynaecology<br>Naaktgeboren, CA ; Julius Centrum voor Gezondheidswetenschappen en Eerstelijns Geneeskunde, University Medical Centre Utrecht<br>van der Post, Joris; Amsterdam UMC, location AMC, Department of obstetrics & gynaecology<br>Bosmans, J; Vrije Universiteit Amsterdam, Department of Health Sciences, Amsterdam Public Health research institute<br>van Kaam, Anton; Amsterdam UMC, location AMC and VUmc, Department of neonatology<br>Schuit, Ewoud; Julius Center for Health Sciences and Primary Care, University Medical Centre Utrecht; Stanford University, Standford Prevention Research Center<br>Mol, Ben; School of Medicine, Monash University, Melbourne, Australia, Department of obstetrics & gynaecology<br>Baalman, Jelle; National Maternity Hospital, Department of obstetrics & gynaecology<br>McAuliffe, Fionnuala; University College Dublin, UCD Perinatal Research Centre; National Maternity Hospital, Department of obstetrics & gynaecology<br>Thornton, Jim; University of Nottingham, Department of obstetrics & gynaecology<br>Kok, Marjolein; Amsterdam UMC, location AMC, Department of obstetrics & gynaecology<br>Oudijk, Martijn; Amsterdam UMC, location AMC, Department of obstetrics & gynaecology
**Primary Subject Heading**:	Obstetrics and gynaecology
Secondary Subject Heading:	Obstetrics and gynaecology
Keywords:	preterm birth, preterm labour, tocolysis, atosiban, perinatal outcome

For peer review only - http://bmjopen.bmj.com/site/about/guidelines.xhtml

Study protocol for a randomised trial for atosiban versus placebo in threatened preterm birth: the APOSTEL 8 study

J. Klumper,[1] W. Breebaart,[1] C. Roos,[1] C.A. Naaktgeboren,[2] J.A.M van der Post,[1] J.E. Bosmans,[3] A.H. van Kaam,[4] E. Schuit,[2] B.W. Mol,[5] J.H. Baalman,[6] F.M. McAuliffe,[6] J.G. Thornton,[7] M. Kok[1] and M.A. Oudijk.[1]

Affiliations

[1]Department of Obstetrics and Gynaecology, Amsterdam UMC, University of Amsterdam, The Netherlands
[2]Julius Centre for Health Sciences and Primary Care, University Medical Centre Utrecht, Utrecht University, the Netherlands
[3]Department of Health Sciences, Faculty of Science, Vrije Universiteit Amsterdam, Amsterdam Public Health research institute, The Netherlands
[4]Department of Neonatology, Amsterdam UMC, Vrije Universiteit van Amsterdam, University of Amsterdam, the Netherlands.
[5]The Robinson Institute, School of Paediatrics and Reproductive Health, University of Adelaide, Australia.
[6]UCD Perinatal Research Centre, School of Medicine, University College Dublin, National Maternity Hospital, Dublin, Ireland
[7]Department of Child Health Obstetrics and Gynaecology, Nottingham University Hospitals NHS Trust, University of Nottingham, United Kingdom

Corresponding author
M.A. Oudijk, MD, PhD
Email: m.a.oudijk@amsterdamumc.nl
Amsterdam University Medical Centres, location AMC
PO box 22660 | 1100 DD Amsterdam
Telephone: +31 20 5667204

J. Klumper: j.klumper@amc.uva.nl
W. Breebaart: breebaart@gmail.com
C. Roos: c.dijkstra@amc.nl
C.A. Naaktgeboren: c.naaktgeboren@umcutrecht.nl
J.A.M van der Post: j.a.vanderpost@amc.uva.nl
J.E. Bosmans: j.e.bosmans@vu.nl
A.H. van Kaam: a.h.vankaam@amc.uva.nl
E. Schuit: e.schuit@umcutrecht.nl
B.W. Mol: ben.mol@monash.edu
J.H. Baalman: jelle.baalman@gmail.com
F.M. McAuliffe: fionnuala.mcauliffe@ucd.ie
J.G. Thornton: jim.thornton@nottingham.ac.uk
M. Kok: m.kok@amc.uva.nl

For peer review only

Abstract

Introduction: Preterm birth complicates more than 15 million pregnancies annually worldwide. In many countries, women who present with signs of preterm labour are treated with tocolytics for 48 hours. Although this delays birth, it has never been shown to improve neonatal outcome. In 2015 the WHO stated that the use of tocolytics should be reconsidered and that large placebo controlled studies to evaluate the effectiveness of tocolytics are urgently needed.

Methods and analysis: We designed an international, multicentre, randomised, double blinded, placebo-controlled clinical trial. Women with threatened preterm birth (gestational age 30 – 34 weeks), defined as uterine contractions with
1) a cervical length of ≤ 15 mm or 2) a cervical length of 15-30 mm and a positive fibronectin test or 3) in centres where cervical length measurement is not part of the local protocol: a positive fibronectin test or insulin-like growth factor binding protein-1 (Actim-Partus test) or 4) ruptured membranes, will be randomly allocated to treatment with atosiban or placebo for 48 hours**.** The primary outcome is a composite of perinatal mortality and severe neonatal morbidity. Analysis will be by intention to treat. A sample size of 1514 participants (757 per group) will detect a reduction in adverse neonatal outcome from 10% to 6% (alpha 0.05, beta 0.2). A cost-effectiveness analysis will be performed from a societal perspective.

Ethics and dissemination: This study has been approved by the Research Ethics Committee (REC) of the Amsterdam University Medical Centres, location AMC, as well as the REC's in Dublin and the United Kingdom. The results will be presented at conferences and published in a peer-reviewed journal. Participants will be informed about the results.

Trial registration number: NTR6646 (date registration 24-aug-2017)

Protocol version: 2.0, dated 27-02-2019

Keywords: preterm birth, preterm labour, tocolysis, atosiban, perinatal outcome

Article summary

Strengths and limitations of this study:

- The primary outcome is perinatal mortality and neonatal morbidity, not prolongation of pregnancy.
- This is the largest randomised trial comparing atosiban to placebo for women with threatened preterm birth.

For peer review only

- Over 40 hospitals in Europe will participate.
- Tocolysis is incorporated in daily routine as it has been the recommendation in many guidelines.
- It will prove to be a challenge in counselling patients to participate in a placebo controlled trial, especially in an acute setting.

Introduction

Preterm birth, defined as birth before 37 weeks gestation, is a major contributor to perinatal mortality and morbidity, complicating over 15 million pregnancies worldwide.[1,2] Of all infant deaths before the age of 5 years, more than one third can be attributed to preterm birth.[3] In addition, spontaneous preterm birth is the leading cause of neonatal morbidity, mostly due to respiratory immaturity, intracranial haemorrhage and infections.[4,5] These conditions can have long-term neurodevelopmental sequelae such as cognitive impairment, cerebral palsy and visual and hearing deficiencies. Preterm birth is one of the largest single contributors to the Global Burden of Disease because of the high mortality early in life and the morbidity of lifelong impairment.[6]

Maternal administration of corticosteroids to accelerate fetal lung maturation is an effective treatment for women with threatened preterm birth.[7] Since steroids have their maximum effect if birth is delayed by 48 hours, many obstetricians administer a tocolytic drug alongside the steroids to allow maximal steroid effect and facilitate transport of the mother to a centre with neonatal intensive care unit facilities if needed. Several tocolytics are used, including β adrenoceptor agonists, cyclooxygenase inhibitors (COX), magnesium sulphate, calcium-channel blockers and oxytocin receptor antagonists. Though more or less effective in delaying delivery, no tocolytics used in obstetrical practice are proven effective in reducing neonatal morbidity and mortality.[8,9] None of the studies so far have been powered to show such an effect.

The two most commonly used tocolytic drugs, atosiban and nifedipine, showed comparable perinatal outcome in the APOSTEL III study.[10] However, neonatal mortality was higher in the nifedipine group, although not significant (5.4% vs. 2.4% RR 2.20; 95% CI 0.91-5.33).

The oxytocin receptor antagonist atosiban has fewer maternal side effects in head to head comparison with alternative drugs,[11] and showed similar effectiveness in delaying birth compared to ritodrine.[12] In placebo controlled trials, a Cochrane review showed that atosiban did not reduce perinatal mortality (RR 2.25, 95% CI 0.79 to 6.38; two studies with 729 infants) or major neonatal morbidity[13], although the quality of this review has been questioned.[14]

For peer review only

One explanation might be that since spontaneous preterm birth is associated in 40-70% of cases with chorioamnionitis,[15,16] tocolysis may prolong fetal exposure to an infectious environment, which may worsen neonatal outcome.

Perinatal outcome has also markedly improved over the last few decades, in part due to postnatal interventions such as exogenous surfactant treatment which reduces mortality and respiratory morbidity in preterm infants.[17] This might also limit the potential benefit of tocolytics.

Worldwide, practice varies widely. Several large institutions in countries like Canada, Scotland and Ireland, rarely use tocolytics, while in the USA, cyclooxygenase inhibitors (indomethacin) and calcium channel blockers (nifedipine) are popular. In Europe, nifedipine and the oxytocin antagonist, atosiban, are both widely used.

In conclusion, current widespread use of tocolytic drugs for this indication is not supported by the available evidence. The primary goal of tocolysis should not be prolongation of pregnancy, but improvement of neonatal outcome. This view is supported by the WHO, as they state in their 2015 guidelines on preterm birth that the effectiveness of tocolytics is not proven, and that placebo controlled studies are urgently needed.[18] Based on the results of the APOSTEL III study,[10] the associated editorial,[19] and its safety profile we chose to evaluate atosiban in the APOSTEL 8 study.

Objective

To test the hypothesis that tocolysis with atosiban in late preterm birth (30 to 34 weeks) reduces neonatal mortality and morbidity and is cost-effective compared with placebo.

Methods and analysis

Design and setting

We will conduct an international, multicentre, double-blind, randomised, placebo-controlled clinical trial, performed in The Netherlands, United Kingdom and Ireland.

Participants/eligibility criteria

Women, aged ≥18 years, with threatened preterm birth and a gestational age between 30 $^{+0}$ and 33 $^{+6}$ weeks are eligible. Threatened preterm birth is defined as uterine contractions with 1) a cervical length of ≤ 15 mm or

For peer review only

2) a cervical length of 15-30 mm and a positive fibronectin test or
3) Or in centres where cervical length measurement is not part of the local protocol: a positive fibronectin test or insulin-like growth factor binding protein-1 (Actim-Partus test) or
4) ruptured membranes.

These inclusion criteria are based on the results and conclusions of the APOSTEL I study[20] and current guidelines within the Netherlands and the United Kingdom. Moreover, our previous APOSTEL III study, with resembling inclusion criteria, showed that half of the women with these criteria deliver within seven days,[10] validating this definition of women at high risk for preterm birth. In addition, the sample size of expected adverse neonatal outcome in the gestational age group of 30-34 weeks, was calculated from the APOSTEL III study.

This study was designed in a pragmatic fashion, in order for the results to be applicable in the current clinical practice. As most national guidelines and local protocols propose treatment for threatened preterm birth in both singleton and multiple pregnancies, as well as women with ruptured membranes, all these categories of patients are eligible for the study.

Women with a contra-indication for tocolysis, signs of fetal distress, clinical signs of intra-uterine infection, previous treatment for threatened preterm birth with corticosteroids in the current pregnancy and known fetal chromosomal or severe structural abnormalities are not eligible.

Procedures, recruitment, randomization and collection of data

Potential participants will be identified by the local research co-ordinators and/or the staff of participating hospitals. Women eligible for the trial will be counselled by doctors, midwifes or research nurses trained in 'good clinical practice', and will be given a patient information form to read. Those who wish to participate, will be asked to give for written informed consent, and are registered within the central trial database. Randomisation will be performed by using sequentially numbered medication packs available in each centre. Only the independent data manager has access to the computer-generated randomization list in which the medication numbers are linked with atosiban or placebo. Treatment allocation is blinded to investigators, participants, clinicians and research coordinators. Randomisation will be balanced with varying block sizes of 2 and 4, and stratified by centre.

At study entry, baseline demographic, past obstetric and medical history will be recorded into the web-based Case Report Form (CRF) accessible through a secure central website (Castor Electronic Data Capture, Ciwit B.V.)[21] Details of delivery, maternal and neonatal

For peer review only

assessments during pregnancy and post-partum period will be recorded on the same system. All data will be coded, processed and stored with adequate precautions to ensure patient confidentially. This is described in a separate data management plan.

Interventions

Participants are allocated to atosiban or matching placebo (0.9% saline) for 48 hours. The medication will be administered by a bolus injection of 6.75 mg/0.9 ml in one minute followed by a continuous infusion of 18 mg/hour for 3 hours followed by a continuous infusion of 6 mg/hour for the remaining 45 hours. Participating women will otherwise be treated according to local protocol based on national guidelines, including corticosteroids $MgSO_4$ for neuroprotection and antibiotics if needed.

Outcome measures

Outcome parameters are in line with the core outcome set for studies on prevention of preterm birth defined by members of GONet and the Core Outcomes in Women's health (CROWN) initiative (www.crown-initiative.org).[22]

The primary outcome measure is a composite of adverse perinatal outcome composed of perinatal in-hospital mortality and six severe perinatal morbidities: bronchopulmonary (BPD), periventricular leucomalacia (PVL) > grade 1, intraventricular haemorrhage > grade 2, necrotizing enterocolitis (NEC) ≥ stage 2, retinopathy of prematurity > grade 2 or needing laser therapy, and culture proven sepsis.

The diagnosis of BPD will be made according to the international consensus guideline as described by Jobe and Bancalari.[23] PVL > grade 1 and intraventricular haemorrhage > grade 2 will be diagnosed by repeated cranial ultrasound according to the guidelines on neuro imaging described by de Vries et al.[24] and Ment et al.[25] NEC ≥ stage 2 will be diagnosed according to Bell.[26] Culture proven sepsis is diagnosed on the combination of clinical signs and positive blood cultures. The components of the composite adverse perinatal outcome will also be assessed separately.

Secondary infant outcomes will be birth within 48 hours, time to birth, gestational age at birth, birth weight, number of days on invasive mechanical ventilation, length of NICU stay, convulsions, asphyxia, meningitis, pneumothorax until hospital discharge.

Maternal outcomes will be mortality, infection of inflammation and harm to mother from interventions (side effects). Side effects are defined as admission to intensive care, anaphylactic shock, dyspnoea, hypotension (leading to CTG abnormalities), liver test

For peer review only - http://bmjopen.bmj.com/site/about/guidelines.xhtml

abnormalities (elevated ASAT or ALAT), general side effects (nausea, vomiting, headache), post-partum haemorrhage defined as > 500 ml blood loss and maternal mortality.

We will ask informed consent to approach the parents for long-term follow-up of the children. We intend, subject to funding, to use standardized questionnaires at 2 years and 5 years of age.

Maternal quality of life will be assessed at randomisation and at three months baby corrected age using the EuroQol (EQ-5D-5L) questionnaire. This consists of five dimensions (mobility, self-care, usual activities, pain/discomfort, anxiety/depression) that are rated using five levels (no problems, slight problems, moderate problems, severe problems, extreme problems).

Societal costs will be assessed using adapted versions of the iMTA Medical and Productivity Cost Questionnaires at three months corrected baby age. Cost data include costs of the intervention, other health care utilization, patient and family costs and costs of productivity losses.

Withdrawal of subjects

Participants can cease study treatment at any time for any reason if they wish to do so. Unless they refuse to allow further data collection, such participants will continue to be followed-up and will be analysed in the group to which they were originally allocated. Participants who decline follow-up will have no further trial data collected. Any results collected up to the point at which they decline follow up will be analysed. Study medication will be discontinued in patients with signs of intra-uterine infections or signs of fetal distress (abnormal CTG, meconium stained amniotic fluid). Data of such participants will continue to be analysed. Further management will be left to the expertise of the responsible clinician. The responsible clinician can contact a perinatologist from the project group in case of suspected side effects or other medical problems. If necessary, treatment will be discontinued.

Monitoring and Safety

An independent data safety monitoring board (DSMB) will focus on both effectiveness and safety. Serious Adverse Events (SAEs) will be collected from the first study-related procedure until 3 months after delivery. All SAEs will be reported to the DSMB within 7 days. Whenever there is proof of effectiveness (at interim analysis) or safety issues (increased (serious) adverse events in one of the two treatment arms) the DSMB will advise whether the trial should be stopped or continued. The data safety monitoring board will be blinded when first analysing the data, but unblinded before reaching a decision.

For peer review only

233 The advice(s) of the DSMB will only be sent to the sponsor of the study. Should the sponsor decide not to fully implement the advice of the DSMB, the sponsor will send the advice to the reviewing Medical Ethics Committee, including a note to substantiate why (part of) the advice of the DSMB will not be followed.

237 A formal interim analysis is planned after data collection of 500 and of 1000 women. At these interim analyses, the Haybittle-Peto alpha spending function will be used, which means that an effect at interim with a p-value <0.001 is considered statistically significant.

Sample size

Based on the APOSTEL III data, the proportion of adverse perinatal outcome in women randomized between 30 and 34 weeks gestation and treated with atosiban was 6%.[10] To show a 40% reduction (10% in the placebo group to 6% in the atosiban group) we need to randomise 1438 women (beta-error 0.2; alpha error 0.05). Assuming a 5% drop-out or loss-to follow up rate, we will randomize 1514 women (757 in each arm).

Statistical analysis

Data analysis

Data analysis will be performed according to the intention-to-treat principle. In the baseline table, categorical variables will be expressed as a number with the percentage of the total allocation arm. Continuous variables will be presented as mean with standard deviation, as geometric mean with 95% confidence interval (CI) or as median with interquartile range, whichever appropriate.

The main outcome 'adverse neonatal outcome' will be assessed on the infant level, using a log-binomial generalized estimating equations model (GEEs) to take into account the correlation of outcomes in multiples, resulting in a relative risk (RR) with accompanying 95% confidence interval (CI). To account for stratified randomization by centre, we will also take centre into the model if the model converges.[27] We will account for interdependence between outcomes in multiple pregnancies by considering the mother as a cluster variable.[28]

The other secondary outcome measures on the child level will be analyzed similarly to the primary outcome measure. Outcomes on the maternal level will be assessed by using a binomial regression model with log-link function. When a statistically significant difference in primary outcome is found between both groups, we will calculate the number needed to treat (NNT). Time to delivery will be evaluated by Kaplan-Meier estimates and Cox proportional hazard analysis, taking into account the different durations of gestation at study entry, and

For peer review only

will be tested with the log rank test. A p-value of less than 0.05 will be considered to indicate statistical significance.

Subgroup analyses

The following subgroup analyses are planned:

1) singleton versus multiple pregnancy,

2) cervical length < 15 mm, versus cervical length 15 - 30 mm and a positive fibronectin test (or no cervical length measurement and a positive Fibronectin test or Partus test),

3) ruptured or unruptured membranes at entry

5) previous preterm birth.

Sensitivity analysis

A sensitivity analysis will be performed excluding multiple pregnancies and pregnancies complicated by preterm premature rupture of membranes.

To assess whether a subgroup effect is present we will add an interaction term between the subgrouping variables and the treatment allocation to the regression model. When an interaction term is statistically significant ($p<0.05$), we will estimate the treatment effect within strata of the subgrouping variable.

Details of the statistical analysis will be describes in separate statistical analysis plan that will be completed before data lock.

Cost-effectiveness analysis

The cost-effectiveness analysis will be done according to the intention-to treat principle. Missing cost and effect data will be imputed using multiple imputation according to the MICE algorithm developed by van Buuren.[29] Rubin's rules will be used to pool the results from the different multiply imputed datasets. Bivariate regression analyses will be used to estimate cost and effect differences between atosiban and placebo while adjusting for confounders if necessary. Incremental cost-effectiveness ratios (ICERs) will be calculated by dividing the difference in the mean total costs between the treatment groups by the difference in mean effect between the treatment groups. Bias-corrected and accelerated bootstrapping with 5000 replications will be used to estimate statistical uncertainty. Uncertainty surrounding ICERs will be graphically presented on cost-effectiveness planes. Cost-effectiveness acceptability curves will be estimated showing the probability that atosiban is cost-effective in comparison with placebo for a range of different ceiling ratios thereby showing decision

For peer review only

uncertainty. Sensitivity analyses will be performed to assess the robustness of the results using different assumptions regarding costs and effects.

Patient and Public Involvement

The preterm birth research line of the Dutch Consortium is in close collaboration with two Dutch patient associations, the VOC (Vereniging van Ouders van Couveusekinderen, freely translated to society of parents of children admitted to NICU) and the NVOM (Nederlandse Vereniging van Ouders van Meerlingen, freely translated to Dutch society of parents of multiples). They are involved in the design of new studies, updated on progress of running trials, and informed of study results. Project members are invited speakers at yearly conferences of these societies to present on the progress of our preterm birth research line. At these conferences, surveys are being performed on patient preferences on study ideas. Tocolysis was deemed an important research issue. Both associations have written support letters to the funding agency ZonMw (The Netherlands organization for health research and development) for the APOSTEL 8 study.

A project panel of parents that experienced a spontaneous preterm birth consisting of 6 couples was involved in the design of our study. A survey was performed during the design of the study amongst members of the closed Facebook group of the VOC, to address questions on whether they would be interested in participation in the APOSTEL 8 study.

The Dutch consortium has a website where it publishes all results of completed studies, and publishes the protocols of currently recruiting studies.

Presentations will be held at yearly conferences at patient organizations and updates on research are being published in the journal of the VOC.

Ethics and dissemination

The Research Ethics Committee (REC) at the Amsterdam University Medical Centres, location AMC, approved this study. Additional regional approval was obtained for the remaining participating hospitals in The Netherlands. Furthermore, the study was approved by the REC of the National Maternity Hospital in Dublin, Ireland, and the REC of East Midlands - Derby in the United Kingdom.

Protocol amendments will be communicated to a relevant parties.

This trial is registered with the Nederlands Trial Register, NTR6646.

A manuscript with the results of the primary study will be published in a peer-reviewed journal. A separate manuscript will be written on the cost effectiveness analysis.

The results of this clinical trial will be presented at conferences and disseminated through publication in a peer-reviewed journal.

For peer review only

Acknowledgements

We would like to thank the collaborators of the study group; the gynecologists of the participating centres for their help as local investigators for the APOSTEL 8 study. We also thank the patient associations VOC and NVOW for their input in this study.

Author contributions

CAN, JAMP, JEB, AHvK, ES, BWM, JHB, FMcA, JGT, MAO, MK were involved in conception and design of the study.

JK and WB drafted the manuscript.

CR, CAN, JAMP, JEB, AHvK, ES, BWM, JHB, FMcA, JGT, MAO, MK reviewed and edited the manuscript. All authors mentioned in the manuscript are member of the APOSTEL study group or collaborators. They participated in the design of the study during several meetings and are local investigators in the participating centres. All authors edited the manuscript and read and approved the final manuscript.

Funding

This study is funded by ZonMw (The Netherlands organization for health research and development), grant number 848041004 and the United Kingdom National Institute for Health Research, Clinical Research Network.

Competing interests statement

JGT received a number of lectures and medical advisory fees from Ferring pharmaceuticals between 2000 and 2016.

References

1. Howson CP, Kinney MV, Lawn JE (editors). Born Too Soon: The Global Action Report on Preterm Birth. Geneva: World Health Organization, 2012
2. Liu L, Johnson HL, Cousens S, Perin J, Scott S, Lawn JE, et al. Global, regional, and national causes of child mortality: an updated systematic analysis for 2010 with time trends since 2000. Lancet 2012;379(9832):2151–61
3. Matthews TJ, Macdorman MF, Thoma ME. Infant Mortality Statistics From the 2013 Period Linked Birth/Infant Death Data Set. Natl Vital Stat Rep. 2015;64(9):1-30

For peer review only

4. Hack M, Fanaroff AA. Outcomes of children of extremely low birthweight and gestational age in the 1990's. Early Hum Dev. 1999;53(3):193-218.
5. Moore T, Hennessy EM, Myles J, et al. Neurological and developmental outcome in extremely preterm children born in England in 1995 and 2006: the EPICure studies. BMJ. 2012;345:e7961.
6. Lozano R, Naghavi M, Foreman K, et al. Global and regional mortality from 235 causes of death for 20 age groups in 1990 and 2010: a systematic analysis for the Global Burden of Disease Study 2010. Lancet. 2012;380(9859):2095-128.
7. Roberts D, Brown J, Medley N, Dalziel SR. Antenatal corticosteroids for accelerating fetal lung maturation for women at risk of preterm birth. Cochrane Database Syst Rev. 2017;3:CD004454.
8. Haas DM, Imperiale TF, Kirkpatrick PR, Klein RW, Zollinger TW, Golichowski AM. Tocolytic therapy: a meta-analysis and decision analysis. Obstet Gynecol 2009; 113: 585–94.
9. Haas DM, Caldwell DM, Kirkpatrick P, McIntosh JJ, Welton NJ. Tocolytic therapy for preterm delivery: systematic review and network meta-analysis. BMJ 2012; 345: e6226.
10. Van Vliet EO, Nijman TA, Schuit E, et al. Nifedipine versus atosiban for threatened preterm birth (APOSTEL III): a multicentre, randomised controlled trial. Lancet. 2016;387(10033):2117-24.
11. De Heus R, Mol BW, Erwich JJ, et al. Adverse drug reactions to tocolytic treatment for preterm labour: prospective cohort study. BMJ. 2009;338:b744.
12. Effectiveness and safety of the oxytocin antagonist atosiban versus beta-adrenergic agonists in the treatment of preterm labour. The Worldwide Atosiban versus Beta-agonists Study Group. BJOG. 2001;108(2):133-42.
13. Flenady V, Reinebrant HE, Liley HG, Tambimuttu EG, Papatsonis DNM. Oxytocin receptor antagonists for inhibiting preterm labour. Cochrane Database of Systematic Reviews 2014, Issue 6. Art. No.: CD004452.
14. Lyndrup J, and Lamont RF The choice of a tocolytic for the treatment of preterm labor: a critical evaluation of nifedipine versus atosiban. Expert Opin. Investig. Drugs (2007) 16(6):843-853.
15. Tita AT, Andrews WW. Diagnosis and management of clinical chorioamnionitis. Clin Perinatol. 2010;37(2):339-54.
16. Romero R, Dey SK, Fisher SJ. Preterm labor: one syndrome, many causes. Science. 2014;345(6198):760-5.
17. Soll R, Ozek E. Prophylactic protein free synthetic surfactant for preventing morbidity and mortality in preterm infants. Cochrane Database Syst Rev. 2010;(1):CD001079.

For peer review only

18. WHO (2015, November 17). WHO recommendation on the use of tocolytic treatment for inhibiting preterm labour. Retrieved from: https://extranet.who.int/rhl/topics/preconception-pregnancy-childbirth-and-postpartum-care/pregnancy-complications/preterm-birth/who-recommendation-use-tocolytic-treatment-inhibiting-preterm-labour
19. Walker KF, Thornton JG. Tocolysis and preterm labour. Lancet. 2016;387(10033):2068-2070.
20. Van Baaren GJ, Vis JY, Wilms FF, et al. Predictive value of cervical length measurement and fibronectin testing in threatened preterm labor. Obstet Gynecol. 2014;123(6):1185-92.
21. Castor: Amsterdam, Ciwit BV the N. Castor electronic data capture. 2017.
22. van 't Hooft J, Duffy JM, Daly M, Williamson P, Meher S, Thom E, et al. A core outcome Set for evaluation of interventions to prevent preterm birth. Obstet Gynecol. 2016;127(1):49–58.
23. Jobe AH, Bancalari E: Bronchopulmonary dysplasia. Am J Respir Crit Care. Med 2001, 163:1723–1729.
24. de Vries LS, Eken P, Dubowitz LM: The spectrum of leukomalacia using cranial ultrasound. Behav Brain Res 1992, 49:1–6.
25. Ment LR, Bada HS, Barnes P, Grant PE, Hirtz D, Papile LA, Pinto-Martin J, Rivkin M, Slovis TL: Practice parameter: neuroimaging of the neonate: report of the Quality Standards Subcommittee of the American Academy of Neurology and the Practice Committee of the Child Neurology Society. Neurology 2002, 58(12):1726–1738.
26. Bell MJ: Neonatal necrotizing enterocolitis. Ann Surg 1978, 187:1–7.
27. Kahan BC. Accounting for centre-effects in multicentre trials with a binary outcome - when, why, and how?. BMC Med Res Methodol. 2014;14:20.
28. Gates S, Brocklehurst P. How should randomised trials including multiple pregnancies be analysed?. BJOG. 2004;111(3):213-9.
29. Van Buuren, S., and Groothuis-Oudshoorn, K. (2011). MICE: Multivariate Imputation by Chained Equations in R. *Journal of Statistical Software* 45(3), 1-68

Word count

3074 words

For peer review only

STANDARD PROTOCOL ITEMS: RECOMMENDATIONS FOR INTERVENTIONAL TRIALS

SPIRIT 2013 Checklist: Recommended items to address in a clinical trial protocol and related documents*

Section/item	Item No	Description	Addressed on page number
**Administrative information**			
Title	1	Descriptive title identifying the study design, population, interventions, and, if applicable, trial acronym	1
Trial registration	2a	Trial identifier and registry name. If not yet registered, name of intended registry	2
	2b	All items from the World Health Organization Trial Registration Data Set	2
Protocol version	3	Date and version identifier	2
Funding	4	Sources and types of financial, material, and other support	11
Roles and responsibilities	5a	Names, affiliations, and roles of protocol contributors	11
	5b	Name and contact information for the trial sponsor	1
	5c	Role of study sponsor and funders, if any, in study design; collection, management, analysis, and interpretation of data; writing of the report; and the decision to submit the report for publication, including whether they will have ultimate authority over any of these activities	11
	5d	Composition, roles, and responsibilities of the coordinating centre, steering committee, endpoint adjudication committee, data management team, and other individuals or groups overseeing the trial, if applicable (see Item 21a for data monitoring committee)	11

For peer review only

Introduction

Background and rationale	6a	Description of research question and justification for undertaking the trial, including summary of relevant studies (published and unpublished) examining benefits and harms for each intervention	3
	6b	Explanation for choice of comparators	4
Objectives	7	Specific objectives or hypotheses	4
Trial design	8	Description of trial design including type of trial (eg, parallel group, crossover, factorial, single group), allocation ratio, and framework (eg, superiority, equivalence, noninferiority, exploratory)	5

Methods: Participants, interventions, and outcomes

Study setting	9	Description of study settings (eg, community clinic, academic hospital) and list of countries where data will be collected. Reference to where list of study sites can be obtained	5
Eligibility criteria	10	Inclusion and exclusion criteria for participants. If applicable, eligibility criteria for study centres and individuals who will perform the interventions (eg, surgeons, psychotherapists)	5
Interventions	11a	Interventions for each group with sufficient detail to allow replication, including how and when they will be administered	6
	11b	Criteria for discontinuing or modifying allocated interventions for a given trial participant (eg, drug dose change in response to harms, participant request, or improving/worsening disease)	7
	11c	Strategies to improve adherence to intervention protocols, and any procedures for monitoring adherence (eg, drug tablet return, laboratory tests)	NA
	11d	Relevant concomitant care and interventions that are permitted or prohibited during the trial	6
Outcomes	12	Primary, secondary, and other outcomes, including the specific measurement variable (eg, systolic blood pressure), analysis metric (eg, change from baseline, final value, time to event), method of aggregation (eg, median, proportion), and time point for each outcome. Explanation of the clinical relevance of chosen efficacy and harm outcomes is strongly recommended	6
Participant timeline	13	Time schedule of enrolment, interventions (including any run-ins and washouts), assessments, and visits for participants. A schematic diagram is highly recommended (see Figure)	6

For peer review only - http://bmjopen.bmj.com/site/about/guidelines.xhtml

Sample size	14	Estimated number of participants needed to achieve study objectives and how it was determined, including clinical and statistical assumptions supporting any sample size calculations	8
Recruitment	15	Strategies for achieving adequate participant enrolment to reach target sample size	5

Methods: Assignment of interventions (for controlled trials)

Allocation:

Sequence generation	16a	Method of generating the allocation sequence (eg, computer-generated random numbers), and list of any factors for stratification. To reduce predictability of a random sequence, details of any planned restriction (eg, blocking) should be provided in a separate document that is unavailable to those who enrol participants or assign interventions	5
Allocation concealment mechanism	16b	Mechanism of implementing the allocation sequence (eg, central telephone; sequentially numbered, opaque, sealed envelopes), describing any steps to conceal the sequence until interventions are assigned	5
Implementation	16c	Who will generate the allocation sequence, who will enrol participants, and who will assign participants to interventions	5
Blinding (masking)	17a	Who will be blinded after assignment to interventions (eg, trial participants, care providers, outcome assessors, data analysts), and how	5
	17b	If blinded, circumstances under which unblinding is permissible, and procedure for revealing a participant's allocated intervention during the trial	5

Methods: Data collection, management, and analysis

Data collection methods	18a	Plans for assessment and collection of outcome, baseline, and other trial data, including any related processes to promote data quality (eg, duplicate measurements, training of assessors) and a description of study instruments (eg, questionnaires, laboratory tests) along with their reliability and validity, if known. Reference to where data collection forms can be found, if not in the protocol	5
	18b	Plans to promote participant retention and complete follow-up, including list of any outcome data to be collected for participants who discontinue or deviate from intervention protocols	7

For peer review only - http://bmjopen.bmj.com/site/about/guidelines.xhtml

Data management	19	Plans for data entry, coding, security, and storage, including any related processes to promote data quality (eg, double data entry; range checks for data values). Reference to where details of data management procedures can be found, if not in the protocol	5, 6
Statistical methods	20a	Statistical methods for analysing primary and secondary outcomes. Reference to where other details of the statistical analysis plan can be found, if not in the protocol	8
	20b	Methods for any additional analyses (eg, subgroup and adjusted analyses)	9
	20c	Definition of analysis population relating to protocol non-adherence (eg, as randomised analysis), and any statistical methods to handle missing data (eg, multiple imputation)	8, 9
**Methods: Monitoring**			
Data monitoring	21a	Composition of data monitoring committee (DMC); summary of its role and reporting structure; statement of whether it is independent from the sponsor and competing interests; and reference to where further details about its charter can be found, if not in the protocol. Alternatively, an explanation of why a DMC is not needed	7, 8
	21b	Description of any interim analyses and stopping guidelines, including who will have access to these interim results and make the final decision to terminate the trial	7, 8
Harms	22	Plans for collecting, assessing, reporting, and managing solicited and spontaneously reported adverse events and other unintended effects of trial interventions or trial conduct	7
Auditing	23	Frequency and procedures for auditing trial conduct, if any, and whether the process will be independent from investigators and the sponsor	7
**Ethics and dissemination**			
Research ethics approval	24	Plans for seeking research ethics committee/institutional review board (REC/IRB) approval	10
Protocol amendments	25	Plans for communicating important protocol modifications (eg, changes to eligibility criteria, outcomes, analyses) to relevant parties (eg, investigators, REC/IRBs, trial participants, trial registries, journals, regulators)	10

For peer review only - http://bmjopen.bmj.com/site/about/guidelines.xhtml

Consent or assent	26a	Who will obtain informed consent or assent from potential trial participants or authorised surrogates, and how (see Item 32)	5
	26b	Additional consent provisions for collection and use of participant data and biological specimens in ancillary studies, if applicable	NA
Confidentiality	27	How personal information about potential and enrolled participants will be collected, shared, and maintained in order to protect confidentiality before, during, and after the trial	5, 6
Declaration of interests	28	Financial and other competing interests for principal investigators for the overall trial and each study site	11
Access to data	29	Statement of who will have access to the final trial dataset, and disclosure of contractual agreements that limit such access for investigators	6
Ancillary and post-trial care	30	Provisions, if any, for ancillary and post-trial care, and for compensation to those who suffer harm from trial participation	Not named
Dissemination policy	31a	Plans for investigators and sponsor to communicate trial results to participants, healthcare professionals, the public, and other relevant groups (eg, via publication, reporting in results databases, or other data sharing arrangements), including any publication restrictions	10
	31b	Authorship eligibility guidelines and any intended use of professional writers	NA
	31c	Plans, if any, for granting public access to the full protocol, participant-level dataset, and statistical code	NA
**Appendices**			
Informed consent materials	32	Model consent form and other related documentation given to participants and authorised surrogates	NA
Biological specimens	33	Plans for collection, laboratory evaluation, and storage of biological specimens for genetic or molecular analysis in the current trial and for future use in ancillary studies, if applicable	NA

*It is strongly recommended that this checklist be read in conjunction with the SPIRIT 2013 Explanation & Elaboration for important clarification on the items. Amendments to the protocol should be tracked and dated. The SPIRIT checklist is copyrighted by the SPIRIT Group under the Creative Commons "Attribution-NonCommercial-NoDerivs 3.0 Unported" license.

For peer review only - http://bmjopen.bmj.com/site/about/guidelines.xhtml
